# A radiosensitizer, gallotannin-rich extract from *Bouea macrophylla* seeds, inhibits radiation-induced epithelial-mesenchymal transition in breast cancer cells

**DOI:** 10.1186/s12906-021-03363-6

**Published:** 2021-07-03

**Authors:** Jiraporn Kantapan, Siwaphon Paksee, Aphidet Duangya, Padchanee Sangthong, Sittiruk Roytrakul, Sucheewin Krobthong, Wipob Suttana, Nathupakorn Dechsupa

**Affiliations:** 1 Molecular Imaging and Therapy Research Unit, Department of Radiologic Technology, Faculty of Associated Medical Sciences, Chiang Mai University, Chiang Mai, 50200 Thailand; 2 Interdisciplinary Program of Biotechnology, Graduate School, Chiang Mai University, Chiang Mai, 50200 Thailand; 3 Department of Chemistry, Faculty of Science, Chiang Mai University, Chiang Mai, 50200 Thailand; 4 Research Center on Chemistry for Development of Health Promoting Products from Northern Resources, Chiang Mai University, Chiang Mai, 50200 Thailand; 5 Functional Ingredients and Food Innovation Research Group, National Center for Genetic Engineering and Biotechnology (BIOTEC), National Science and Technology Development Agency (NSTDA), Pathum Thani, 12120 Thailand; 6 National Omics Center (NOC), National Science and Technology Development Agency (NSTDA), Pathum Thani, 12120 Thailand; 7 Department of Biomedical Science, School of Health Science, Mae Fah Luang University, Chiang Rai, 57100 Thailand

**Keywords:** Radiosensitizer, EMT, Cancer stem cells, Breast cancer, Senescence, Apoptosis, Maprang seed extract, Radiotherapy, Proteomics

## Abstract

**Background:**

Radioresistance can pose a significant obstacle to the effective treatment of breast cancers. Epithelial–mesenchymal transition (EMT) is a critical step in the acquisition of stem cell traits and radioresistance. Here, we investigated whether Maprang seed extract (MPSE), a gallotannin-rich extract of seed from *Bouea macrophylla* Griffith, could inhibit the radiation-induced EMT process and enhance the radiosensitivity of breast cancer cells.

**Methods:**

Breast cancer cells were pre-treated with MPSE before irradiation (IR), the radiosensitizing activity of MPSE was assessed using the colony formation assay. Radiation-induced EMT and stemness phenotype were identified using breast cancer stem cells (CSCs) marker (CD24^−/low^/CD44^+^) and mammosphere formation assay. Cell motility was determined via the wound healing assay and transwell migration. Radiation-induced cell death was assessed via the apoptosis assay and SA-β-galactosidase staining for cellular senescence. CSCs- and EMT-related genes were confirmed by real-time PCR (qPCR) and Western blotting.

**Results:**

Pre-treated with MPSE before irradiation could reduce the clonogenic activity and enhance radiosensitivity of breast cancer cell lines with sensitization enhancement ratios (SERs) of 2.33 and 1.35 for MCF7 and MDA-MB231cells, respectively. Pretreatment of breast cancer cells followed by IR resulted in an increased level of DNA damage maker (γ-H2A histone family member) and enhanced radiation-induced cell death. Irradiation induced EMT process, which displayed a significant EMT phenotype with a down-regulated epithelial marker E-cadherin and up-regulated mesenchymal marker vimentin in comparison with untreated breast cancer cells. Notably, we observed that pretreatment with MPSE attenuated the radiation-induced EMT process and decrease some stemness-like properties characterized by mammosphere formation and the CSC marker. Furthermore, pretreatment with MPSE attenuated the radiation-induced activation of the pro-survival pathway by decrease the expression of phosphorylation of ERK and AKT and sensitized breast cancer cells to radiation.

**Conclusion:**

MPSE enhanced the radiosensitivity of breast cancer cells by enhancing IR-induced DNA damage and cell death, and attenuating the IR-induced EMT process and stemness phenotype via targeting survival pathways PI3K/AKT and MAPK in irradiated breast cancer cells. Our findings describe a novel strategy for increasing the efficacy of radiotherapy for breast cancer patients using a safer and low-cost natural product, MPSE.

**Supplementary Information:**

The online version contains supplementary material available at 10.1186/s12906-021-03363-6.

## Background

Adjuvant radiotherapy following surgery remains a standard therapeutic regimen for achieving loco-regional control and improving overall survival in breast cancer patients [1]. The disease-free and overall survival rates are improved using additional radiotherapy in the breast cancer patients, but the effects are limited by the radioresistance phenomenon. Radioresistance may occur at the beginning of treatment (intrinsic radioresistance) or develop during fractionated radiotherapy treatment (acquired radioresistance) [2, 3]. These radioresistant cells can repopulate the tumor site and then lead to recurrence and treatment failure.

Epithelial–mesenchymal transition (EMT) is a reversible process through which epithelial cells transform into a mesenchymal state characterized by the loss of epithelial features, including expression of E-cadherin and cell-to-cell contact and acquired mesenchymal features such as increased expression of N-cadherin and vimentin, invasion and metastasis, and change in morphology to fibroblast-like cells [4]. EMT was reported to contribute to the dedifferentiation in non-stem cancer cells into cancer stem cells [5]. A study in breast cancers revealed that EMT promotes stemness in breast cancer cells by gaining cancer stem cell properties, including the ability to self-renew and tumorigenicity, and exhibiting a CD44^+^/CD24^−/low^ phenotype [4, 6]. In addition, accumulating evidence is showing that a radiation-induced EMT program is an essential process involved in radioresistance [7, 8]. Altogether, these finding indicate a crucial link amongst metastases, EMT, cancer stem cells, and radioresistance.

The key driving survival pathways, such as the phosphatidylinositol-3-kinase (PI3K)/protein kinase B (AKT) and mitogen-activated protein kinase (MAPK) signaling pathways, are activated in irradiated cancer cells. The activation of these pathways protects cancer cells from the cytotoxic effects of radiation, ultimately leading to the development of radioresistance [9]. The activation of PI3K/AKT is highly involved in the development of radioresistance in several solid tumors including breast, lung, and prostate cancers [10–12]. Therefore, targeting these survival pathways in combination with radiotherapy may enhance therapeutic efficacy in breast cancer by attenuating cellular defense in response to treatment.

We have been investigating the promising anti-cancer activity of *Bouea macrophylla* Griffith extract (MPSE), also known as Maprang or Marian plum, an economic Thai fruit. MPSE has been shown to exert several pharmacological activities including anti-microbial, anti-cancer, and anti-oxidation activities [13]. Our previous studies have identified MPSE contents by high performance liquid chromatography (HPLC) and liquid chromatography/mass spectrometry (LC/MS) analysis revealed that MPSE contains high amounts of active compounds 1,2,3,4–6-pentyl-O-galloyl-β-d-glucose (PGG), ethyl gallate (EG), and gallic acid (GA). Moreover, MPSE also induced cell cycle arrest and apoptosis with a prominent effect on human breast cancer cells than normal mammary epithelial MCF-10A cells [14]. Interestingly, pre-treatment with MPSE before irradiation suppressed the expression of multidrug resistance proteins in the radiation-survived population of MCF7 cells [15]. In particular, PGG has been extensively studied for its anti-cancer properties. PGG has been demonstrated to suppress the functions of estrogen receptor alpha (ERα) and to modulate the ErbB lineage of proteins (ErbB)/PI3K/AKT pathway in hormone-dependent breast cancer cells [16]. PGG also exerts an anti-invasive effect by suppressing the epidermal growth factor receptor (EGFR)/c-Jun N-terminal kinases (JNK) pathway and controlling matrix metalloproteinase-9 (MMP-9) expression, resulting in suppresses bone metastasis in nude mice treated with an intratibial injection of PC-3 prostate cancer cells [17]. Given their diverse anti-cancer properties, we hypothesized that combining MPSE and IR treatment might attenuate the IR-induced survival pathways and thereby enhance the radiation effect against breast cancer cells.

Herein, we investigated whether Maprang seed extract (MPSE), a gallotannin-rich extract of seed from *Bouea macrophylla* Griffith, could inhibit the radiation-induced EMT process and enhance the radiosensitivity of breast cancer cells. This study provides evidence of a molecular mechanism for the radiosensitizer effect of MPSE, demonstrating that MPSE in combination with radiotherapy inhibits the radiation-induced EMT process by attenuating the PI3K/AKT and MAPK pathways and increasing therapeutic efficacy in breast cancer.

## Methods

### Chemicals and reagents

3-(4,5-dimethylthiazol-2-yl) 2,5-diphenyltetrazolium bromide (MTT), bovine serum albumin (BSA), human insulin, epidermal growth factor (EGF), basic fibroblast growth factor ((bFGF), and hydrocortisone were purchased from Millipore Corporation (Bedford, MA, USA). The annexin V-fluorescein isothiocyanate (FITC) apoptosis detection kit and senescence detection kit were also purchased from Millipore Corporation (Bedford, MA, USA). Roswell Park Memorial Institute (RPMI 1640) and Dulbecco’s Modified Eagle’s Medium/Nutrient Mixture F-12 (DMEM/F-12) were purchased from Caisson Lab (Smithfield, UT, USA). Trypsin-EDTA, fetal bovine serum (FBS), penicillin, and streptomycin were purchased from Gibco company (Gibthai, Bangkok, Thailand). Phenylmethylsulfonyl fluoride (PMSF) and cocktail protease inhibitor were purchased from HiMedia Laboratories (Marg, Mumbai, India). Additionally, 4′,6-diamidino-2-phenylindole, dihydrochloride (DAPI) was obtained from Thermo Fisher Scientific (Waltham, MA, USA). Primary antibodies against Zinc finger E-box binding homeobox 1 (ZEB1, Cat. No. ABD53), E-cadherin (Cat. No. MAB3199), vimentin (Cat. No. AB1620), total AKT (Cat. No. 05–796), phosphorylated AKT (Ser473, Cat. No. 05–1003), total JNK/SAPK1(Cat. No. 06–748), phosphorylated JNK (Thr183/Tyr185, Thr221/Tyr223, Cat. No. 07–175), total ERK1/2 (Cat. No. MABS827), and phosphorylated ERK1/2 (Thr202/Tyr204, Thr185/Tyr187, Cat. No. 05-797R), and glyceraldehyde-3-phosphate dehydrogenase (GAPDH, Cat. No. AB2302), horseradish peroxidase labeled secondary antibodies (Goat Anti-Mouse IgG antibody, Cat. No. AP124P and Goat Anti-Rabbit IgG antibody, Cat. No. AP132P) were purchased from Millipore Corporation (Bedford, MA, USA).

### Plant materials and extraction

*Bouea macrophylla* Griffith (Maprang) seeds were collected between March and April 2015 from the Maprang Plantation located in Nakhon Nayok Province. The samples were collected with permission from private landowners and botanically identified by comparison with a voucher specimen (CMUB39942) deposited in the CMUB herbarium at Chiang Mai University, Thailand. The Maprang seed crude extract was prepared, as described previously [13]. In brief, 50 g of the minced dried seeds were macerated in 75% ethanol (500 mL) for 7 days with daily shaking. Then, the extracts were filtered by Kesselguhr and concentrated by a rotatory evaporator (Buchi Rotavapor R-100, Switzerland). After lyophilization, a total of 20 g of extract powder (MPSE) was obtained (40% yield). MPSE was collected and stored at room temperature in a desiccator for further study. To prepare the stock solution, MPSE was solubilized with deionized water (Pure Lap Option-Q, ELGA, UK) at a final concentration of 1 mg/mL and then filtered with a 0.22 μm syringe filter, aliquoted, and stored at − 80 °C until being used. The active ingredients including, penta-O-galloyl-β-D-glucose hydrate (PGG), ethyl gallate (EG), and gallic acid (GA), were quantified by using Shimadzu LC-20 AD Prominence Liquid Chromatograph system equipped with an SPD-M20A Prominence Diode Array Detector (Shimadzu, Japan).

### Cell culture conditions and irradiation treatment

The radioresistant triple-negative breast cancer cell line MDA-MB231 and the more sensitive estrogen receptor (ER+) MCF-7 cancer cell line were used in this study. Human breast cancer cell lines MCF7 and MDA-MB231 were purchased from American Type Culture Collection (ATCC® HTB-22™, ATCC® HTB-26™, Manassas, VA, USA). Cells were cultured with RPMI 1640 supplemented with 10% fetal bovine serum (FBS) and 1% penicillin-streptomycin in a 95% air humidified atmosphere and 5% CO_2_ at 37 °C. Fractionated irradiation consisting of 3.3 Gray (Gy)/day for 3 days or a single doses of IR was performed on cells seeded in T75 cm^2^ cell culture flask in 12 mL of completed medium in a linear 6 MV X-ray accelerator (Primus, Siemens Healthineers, Malvern, PA, USA) at a dose rate 200 MU/minute.

### Clonogenic assay

Briefly, cells were cultured for 24 h to form a monolayer in T75 cm^2^ cell culture flask. After exposure to 2–8 Gy X-ray, cells were trypsinized and plated in a 6-well plate. Cells were maintained for 14 days to allow colonies to form. Colonies of cancer cell were determined as a group that consisted of at least 50 cells. After that, colonies were fixed in fixation solution (3:1 of methanol: acetic acid) for 30 min at room temperature, then stained with 0.5% crystal violet in methanol for 30 min at room temperature in total darkness. After finishing the staining, the colonies were counted under a microscope. These data were used for survival curve plotting and fitting as a function of X-ray dosage using a linear-quadratic model using OriginPro 8 software (Northampton, MA, USA).

### Spheroid formation assay

The mammosphere assay was performed using Geltrex® LDEV-Free Reduced Growth Factor Basement Membrane Matrix (Thermo Fisher Scientific, Waltham, MA, USA) following the manufacturer’s guidelines. Briefly, breast cancer cells (5 × 10^3^ cells) were seeded to each well of a 96-well plate containing Geltrex® Matrix with serum-free DMEM/F12 supplemented with 1% BSA, 5 μg/mL insulin, 25 ng/mL bFGF, 25 ng/mL EGF, 0.5 μg/mL hydrocortisone, and B-27 supplement. Cells were incubated for 14 days to form mammospheres. Mammosphere pictures were captured with an inverted microscope (Nikon, ECLIPSE Ts2, Tokyo, Japan) and the number of mammospheres was counted.

### Soft agar colony assay

MCF7 cells were treated with either IR alone or MPSE combined with 6 Gy IR. Cells were harvested by trypsinization at 48 h after treatment. The harvested cells were suspended in 0.3% agarose with RPMI 1640 medium containing 10% FBS at a density of 5 × 10^3^ cells and seeded in a 6-well plate coated with base layer of 0.5% agar. Cells were incubated at 37 °C for 2 weeks, and colony formation was determined at the end using a phase-contrast microscope (Nikon, ECLIPSE Ts2, Tokyo, Japan). The colony numbers of colonies larger than 20 μm were counted using ImageJ software (Bethesda, Maryland, USA).

### Detection of breast cancer stem cells surface markers

To detect cell surface markers, CD44-FITC- and CD24-PE-conjugated dyes were used for analysis. Cells (2 × 10^5^) were detached by trypsin and washed with phosphate buffer saline (PBS). Next, 1% BSA in PBS was added together with FITC-conjugated anti-CD44 or PE-conjugated anti-CD24 antibodies. The solution was incubated at 4 °C for 30 min and then flow cytometry was performed (BECKMAN COULTER, Epics XL-MCL, Brea, CA, USA.) Flow cytometric data were then analyzed using FlowJo10 software (Vancouver, BC).

### Wound healing assay

Wound healing assays to assess cell migration were conducted in 6-well plates. Briefly, cells were seeded in 6-well plates and grown to 80–90% confluence. The space was created by slowly scratching the monolayer with a 200 μL sterile tip across the center of the well. The well was then gently washed twice with RPMI 1640 to remove the detached cells and replenished with fresh medium. Wound closure was monitored and photographed at 3, 6, 12, and 24 h with an inverted microscope (Nikon, ECLIPSE Ts2, Tokyo, Japan). To quantify the migrated cells, pictures of the initial wounded monolayers were compared with the corresponding pictures of cells at the end of the incubation. The width of the space was analyzed using Image J software (Bethesda, Maryland, USA) to determine the percentage of wound closure.

### Transwell migration assay

To assess cell motility, Boyden chamber assays were conducted in 24-well plates with a Cell Culture Insert Chamber (Corning Life Sciences, MA, USA). Cells were serum-starved for 24 h before being seeded in the top chamber of the transwell set-up. Then, the completed culture media containing FBS were added to the bottom chamber. Cells were grown in an incubator for 24 h, then non-migrated cells in the upper chamber were scraped and washed with PBS. Invaded cells were stained with crystal violet and pictures were taken with Nikon inverted microscope (Nikon, ECLIPSE Ts2, Tokyo, Japan). Migrated cells were quantified using ImageJ software (Bethesda, Maryland, USA).

### Apoptosis assay

Cells were seeded into a 6-well plate for 24 h to allow attachment before being treated with either MPSE, IR alone, or MPSE combined with 6 Gy IR X-ray. Cells were harvested by trypsinization at 48 h after treatment. The harvested cells were stained using annexin V-FITC apoptosis detection kit (Millipore Corporation, Bedford, MA, USA) following the manufacturer’s instructions. Briefly, the harvested cells were washed by 1× binding buffer, then centrifuged at 7000 rpm for 1 min and the supernatant was removed. The pellets were stained by adding 1× binding buffer, annexin V-FITC, and propidium iodide (PI) for 20 min at room temperature in total darkness. The stained cells were immediately analyzed by flow cytometry (BECKMAN COULTER, Epics XL-MCL, Brea, CA, USA). Flow cytometric data were then analyzed by FlowJo10 software (BD, USA).

### Senescence-associated-β-galactosidase (SA-β-gal) staining for cellular senescence

Cells were seeded into 6-well plates for 24 h to allow attachment before being treated with either MPSE, IR alone, or MPSE combined with 6 Gy IR X-ray. Cells were stained using a senescence cells histochemical staining kit (Millipore Corporation, Bedford, MA, USA) following the manufacturer’s instructions at day 6 after irradiation. Briefly, cells were washed twice by PBS 1× before adding 1× fixation buffer, then incubating at room temperature for 6 min. The fixation buffer was removed, then rinsed off three times with PBS 1×. Cells were stained using a staining mixture and then incubated at 37 °C without CO_2_ for 2 h. The cells were observed under a microscope (Nikon, ECLIPSE Ts2, Tokyo, Japan) at a 200× magnification and the percentages of blue-stained cells (senescent cells) were calculated.

### γH2AX assay

Cells were plated in chamber slides and pre-treated with MPSE 24 h before irradiation with 6 Gy X-ray. At 48 h post-irradiation, the cells were fixed with 4% paraformaldehyde for 15 min at room temperature. Then, cells were permeabilized with 0.3% TritonX-100 and blocked with 5% BSA in PBS solution at room temperature for 30 min. The cells were then incubated overnight at 4 °C with primary rabbit polyclonal antibodies against γH2AX (1:500). After primary antibody incubation, the cells were washed with PBS with Tween 20 (PBST) and incubated with 1:200 diluted Alexa Fluor 488 conjugated rabbit anti-human IgG antibodies (Invitrogen, Thermo Fisher Scientific, Waltham, MA, USA) for 1 h at room temperature. Nuclei were counterstained with 4′,6-diamidino-2-phenylindole, dihydrochloride (DAPI, 1 μg/mL). Pictures were captures with an inverted fluorescence microscope (Nikon, ECLIPSE Ts2,Tokyo, Japan). γH2AX intensity was quantified using ImageJ software (Bethesda, Maryland, USA).

### Gene expression

The total RNA was extracted with an E.Z.N.A.® Total RNA Kit I (OMEGA bio-tek, Norcross, GA USA) and reverse transcription reactions were performed using the ReverTra Ace® qPCR RT Master Mix with gDNA Remover kit (TOYOBO, Osaka, Japan) following the manufacturer’s guidelines. The expressions of seven genes, including *CDH1, VIM, ZEB1, Nanog, Sox2,* and *Oct4*, were determined by quantitative PCR (qPCR), and *HPRT* was used as a housekeeping gene [18]. The qPCR reactions were performed in triplicate using Luna® Universal qPCR Master Mix (New England Biolabs, UK) with 100 ng cDNA template and 0.4 μM of target-specific primers in an Exicycler™96 Bioneer machine (Bioneer Corporation, Daejoen, Korea). The qPCR reaction used the following conditions: initial denaturation at 95 °C for 90 s and then denaturation at 95 °C for 20 s, annealing at 51 °C for 15 s, and extension at 60 °C for 30 s for 45 cycles. The expression of the genes was normalized against *HPRT* and the relative genes expression level was calculated from the C_t_ value [19] (the raw data C_t_ value are available in Table S4). The primer sequences are shown in Table S1.

### Liquid chromatography - tandem mass spectrometry (LC-MS/MS)

The cells were disrupted by lysis buffer cocktail (0.5% Triton X-100, 10 mM dithiothreitol (DTT) in 20 mM HEPES-NaOH, pH 8.0) supplemented with protease inhibitor cocktail (2 mM PMSF, 0.3 μM Aprotinin, 0.3 μM, 2 mM EDTA, and 10 μM E-64). Proteolytic digestion by using 500 ng of sequencing grade trypsin (Promega, Walldorf, Germany) was added to the protein solution and incubated at 37 °C for 3 h. The peptides were cleaned-up by C18-ZipTip, dried and resolubilized in 0.1% formic acid. The protonated peptide solutions were analyzed with an Impact II UHR-TOF MS System (Bruker Daltonics Ltd., Germany) coupled to a nanoLC system: UltiMate 3000 LC System (Thermo Fisher Scientific, Waltham, MA, USA) equipped with a nano captive spray ion source. The mass spectrometry was operated in positive ion mode over the range (m/z) of 150–2200 (Compass 1.9 for otofSeries software, Bruker Daltonics). Mass accuracy (TOF detector) calibrated with LC/MS tuning mix for ESI (Agilent, USA) was within 1.6 ppm. The raw LC-MS runs were conducted using CompassXport Version 3.0.9.2 (Bruker Daltonics GmbH, Germany) to convert the raw spectra (.d file format) to mzXML data format.

### Bioinformatics and data analysis

The mzXML of LC-MS/MS datasets for label-free quantification were evaluated for relative comparison of peak intensities of each peptide ion in all datasets of LC-runs using DeCyder MS Differential Analysis software [20]. Peak detection (signal/noise ratio < 2) and peak matching (peptide ion peak alignment) were performed by the Batch and Pepmatch module in the DeCyder MS Differential Analysis software. All dataset of MS/MS peak lists from the Pepmatch module in the DeCyder MS Differential Analysis software were exported to Mascot generic format (.mgf file format) and submitted for in-house database search using the Mascot software (Matrix Science, London, U.K.). The dataset of MS/MS peak lists was searched against *Homo sapiens* (Uniprot database) for protein identification by following these parameters: trypsin as digesting enzyme, maximum of 3 missed cleavages, MS peptide tolerance of 1.0 Da, MS/MS peptide tolerance of 0.5 Da, carbamidomethylation modification of cysteine residue as fixed modification, and the oxidation of methionine and acetylation of the protein N-terminus as variable modifications. Only significant peptides with ANOVA values lower than 0.05 were accepted. The gene list enrichment analysis to predict pathways and gene ontology (GO) was conducted using Enrichr software (https://maayanlab.cloud/Enrichr/) [21]. Functional analysis of differential expressed proteins revealed their associated biological processes. A Venn diagram (http://bioinformatics.psb.ugent.be/webtools/Venn/) was used to show the differences between proteins lists originating from different differential analyses [22]. The STRING software version 11 (https://string-db.org/) search tool was used for protein–protein interaction analysis to examine the functional interaction networks between the identified proteins [23]. The heatmap visualization was conducted using OriginPro 2018 software (Northampton, MA, USA).

### Western blot analysis

Following treatment, cells were lysed with lysis buffer containing 8 M urea, 2 M thiourea, 20 mM dithiothreitol (DTT), 4% 3-[(3-Cholamidopropyl)dimethyl-ammonio]-1-propane sulfonate (CHAPS), 1 mM phenylmethylsulfonyl fluoride (PMSF), and cocktail protease inhibitor mixture for 30 min on ice. The protein contents were evaluated by Bradford assay (Millipore Corporation, Bedford, MA, USA). Samples with equal amounts of protein (15 μg) were resolved by SDS- polyacrylamide gels electrophoresis and transferred onto polyvinyl difluoride (PVDF) membrane (Millipore Corporation, Bedford, MA, USA). Transferred membranes were blocked for 1 h in 5% non-fat dry milk in Tris-buffered saline with Tween and incubated overnight with specific primary antibodies against E-cadherin, vimentin, ZEB1, total AKT, phosphorylated AKT, total JNK, phosphorylated JNK, total ERK1/2, phosphorylated ERK1/2, and GAPDH. Membranes were washed three times with Tris-buffered saline and Tween 20 and incubated with appropriate horseradish peroxidase labeled secondary antibodies for 1 h at room temperature. The blot was detected by Clarity and Clarity Max ECL Western Blotting Substrates (Bio-Rad Laboratories, Singapore), and the signal was exposed with X-ray film. The images were scanned, and the intensity of each band was captured using an Image master 2-Dimention platinum version 5.0 (GE Healthcare Amersham Bioscience, UK).

### Statistical analysis

Data are expressed as mean ± standard deviations (SDs) of at least three independent experiments. The differences in the mean values were determined by one-way ANOVA followed by Tukey’s HSD test using IBM® SPSS® Statistics Subscription software (IBM Corp. in Armonk, NY, USA). A value of *p* < 0.05 was considered statistically significant.

## Results

### MPSE radio-sensitizes breast cancer cells

**Fig. 1:**
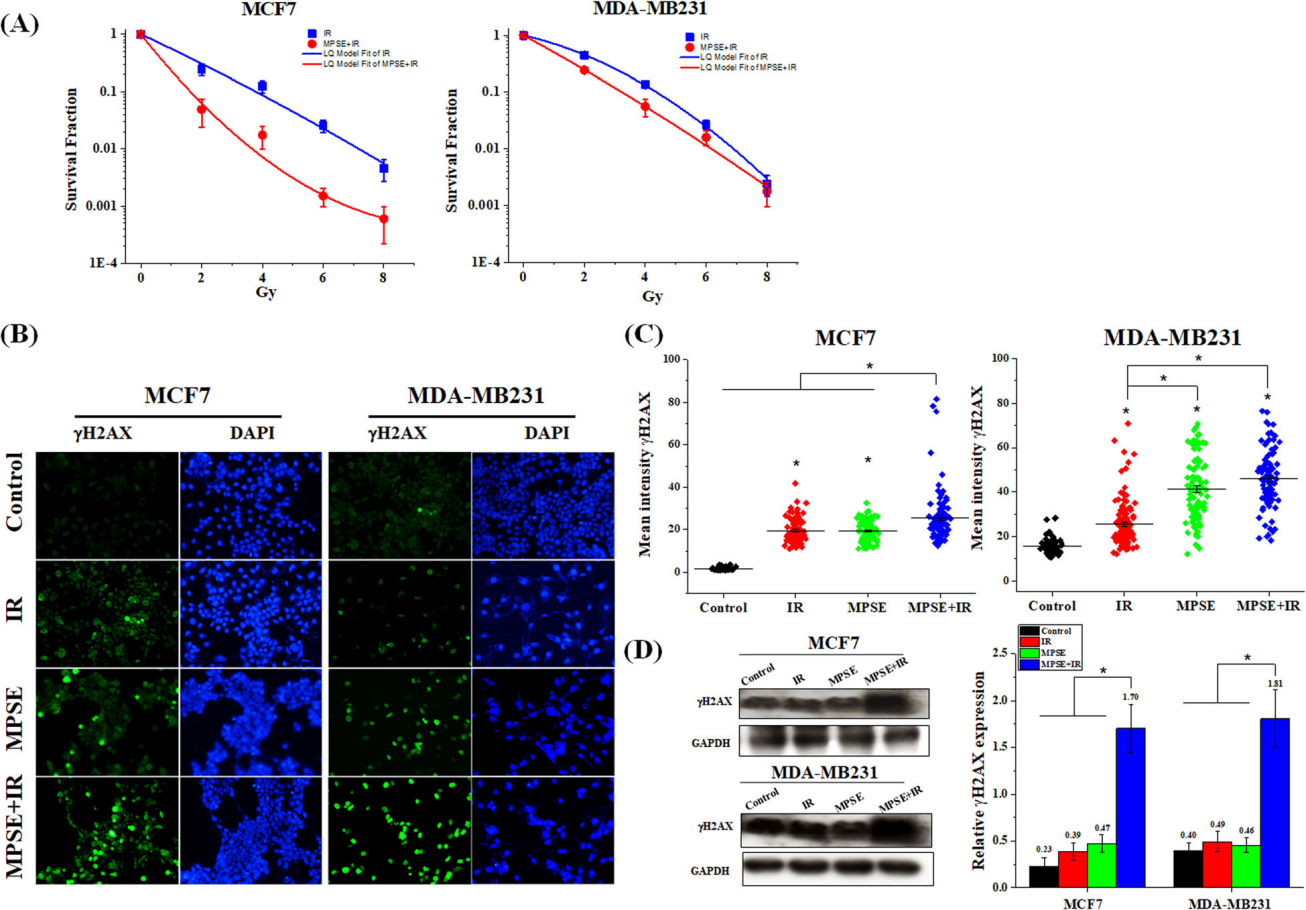
Maprang seed extract (MPSE) enhances the radiosensitivity of breast cancer cells. **A** MCF7 and MDA-MB231 cells were pre-treated with 20 μg/mL MPSE for 24 h and then subjected to increasing doses of ionizing radiation (IR); the survival fraction of MCF7 and MDA-MB231 cells at 14 days post-treatment were determined by clonogenic assay. **B** Representative fluorescence images of γH2AX foci. MCF7 and MDA-MB231 cells were grown on glass coverslips and pre-treated with MPSE for 24 h and then irradiated with 6 Gy of IR. After 24 h, cells were analyzed for γH2AX foci by immunostaining with anti-γH2AX. **C** Quantitative assessment of mean fluorescence intensity values of γH2AX in breast cancer cells. More than 100 cells were randomly picked for quantification. Results are presented as mean ± SD. The statistical significance of each group was analyzed by a one-way ANOVA and Tukey’s HSD test (* p < 0.05*.*) **D** Western blotting analysis with anti-γH2AX and anti-GAPDH antibodies on breast cancer cells treated with IR and MPSE, alone or in combination. The immunoblot signal intensities were quantified by densitometry; the mean data from each experiment were normalized to the GAPDH. Three independent experiments were performed. The results are presented as mean ± SD (numbers above the bars represent the mean of each group). The statistical significance of each group was analyzed by a one-way ANOVA and Tukey’s HSD test (* p < 0.05)

To examine whether MPSE radio-sensitizes breast cancer cells, we performed a clonogenic survival assay by pre-treating breast cancer MCF7 and MDA-MB231 cells with 20 μg/mL of MPSE for 24 h prior to irradiation with 2, 4, 6, or 8 Gy of IR. As shown in Fig. 1A, whereas IR exposure alone induced a small reduction in the survival fraction in both MCF7 and MDA-MB231 cells, pre-treatment with MPSE significantly decreased the clonogenic survival of both MCF7 and MDA-MB231 cells. The sensitization enhancement ratios (SERs) at a survival fraction level of 0.10 (SF0.1) were 2.33 and 1.35 for MCF7 and MDA-MB231 cells, respectively, after values were normalized to account for the effect of MPSE alone. Notably, MCF7 was more sensitive to the combined treatment of IR with MPSE in comparison to MDA-MB231 cells. To understand the molecular mechanism of MPSE-induced sensitizing breast cancer cells to radiation, MCF7- and MDA-MB231-treated cells were evaluated for IR-induced DNA damage. The results revealed that the number of phosphorylated histone H2AX (γH2AX) foci increased in both MCF7 and MDA-MB231 cells treated with IR compared with untreated control cells (Fig. 1B). Quantitative fluorescence analysis showed that the total fluorescence intensity of γH2AX markedly increased in IR-treated MCF7 and MDA-MB231 compared to the control. MPSE pre-treatment resulted in a further increase in the fluorescence intensity of γH2AX, suggesting that MPSE in combination with IR produces more DNA damage in breast cancer cells (Fig. 1B,C). We further investigated the expression level of phosphorylated histone H2AX (γH2AX) in breast cancer cells. As shown in Fig. 1D, radiation-induced γH2AX formation occurred in both breast cancer cell lines and the combination of MPSE further increased the expression level of γH2AX. These observations suggested that MPSE pre-treatment could increase the extent of DNA damage induced by radiation and enhance the radiosensitivity of breast cancer cells.

### MPSE pre-treatment combined with IR more effectively induces death of breast cancer cells

**Fig. 2:**
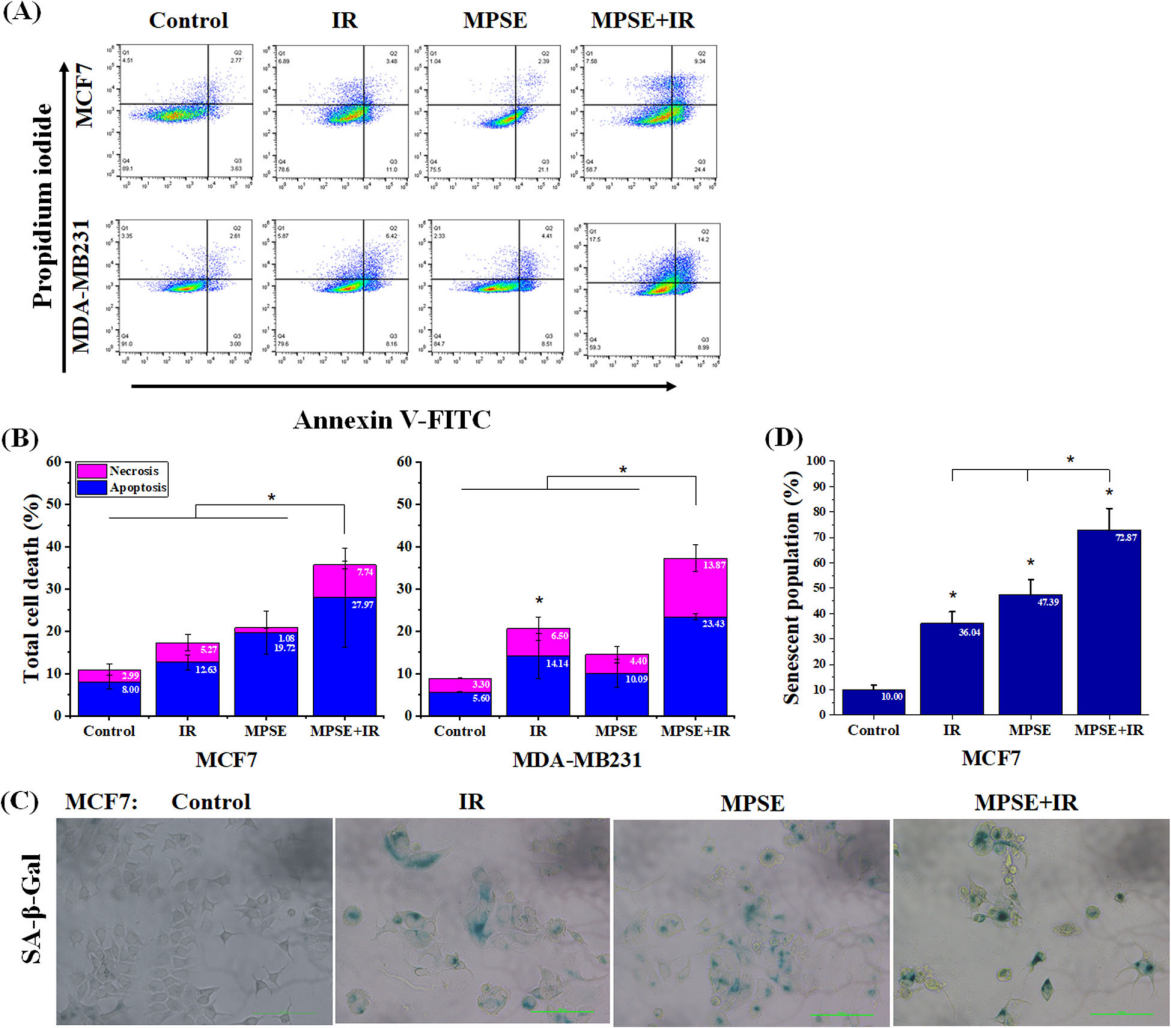
Combination therapy with MPSE enhances IR-induced cell death in breast cancer cells. **A** Apoptosis was assessed by flow cytometry with FITC-conjugated annexin V/PI staining. MCF7 and MDA-MB231 cells were pre-treated with MPSE (20 μg/mL) for 24 h, and then irradiated with 6 Gy of IR. After 24 h of incubation, MCF7 and MDA-MB231 cells were co-stained with annexin V-FITC and PI and were subjected to flow cytometer. **B** Quantification of apoptotic cell percentage showed that MPSE enhanced IR-induced apoptosis. The data are presented as mean ± SD of three independent experiments (numbers in the bars represent the mean of each group). The statistical significance of each group was analyzed by a one-way ANOVA and Tukey’s HSD test (* p < 0.05). **C** Representative image of senescent cells (blue-stained cells) taken from inverted microscope at a 200× magnification. **D** The percentage of senescent cell population of breast cancer cell lines after sixth day of irradiation. Bar graph shows a mean ± SD (numbers in the bars represent the mean of each group). The statistical significance of each group was analyzed by a one-way ANOVA and Tukey’s HSD test (* p < 0.05)

To further explore the mechanisms of MPSE-induced sensitization of breast cancer cells to radiation, we investigated whether MPSE could promote IR-induced apoptosis. Radiation-induced apoptosis in breast cancer cell lines was quantitatively determined using Annexin-V/FITC and propidium iodide staining. The combined effect of MPSE and IR was evaluated after 48 h of treatment. As show in Fig. 2A,B, the combined treatment of MPSE and IR showed a markedly increased apoptotic effect in both breast cancer cell lines compared with single treatment by IR or MPSE, with a more pronounced effect in MCF7 cells. A higher number of apoptotic cells was observed in the combined treatment group (27.97%) than the IR-alone group (12.63%). Like the effects observed in MCF7 cells, the combined treatment led to the apoptotic induction in MDA-MB-231 cells with a more noticeable impact on necrotic cell death led to higher total cell death rate than a single treatment.

The analysis of senescence based on cellular β-galactosidase (SA-β-Gal) activity 6 days after irradiation with 6 Gy revealed that IR induces a senescent response in MCF7 cells, as we observed an increase in β-galactosidase staining and senescence-associated cell morphology alterations compared with the untreated control (Fig. 2C). The combination treatment significantly increased the senescent effect in MCF7 breast cancer cell lines compared with the single treatment of IR or MPSE. Figure 2D shows that the percentage of senescent cells in the combined treatment was approximately two-fold that of the single treatment. These results indicated that either IR or the combination of MPSE and IR effectively induces cellular senescence rather than apoptotic cell death in MCF7 cells. Therefore, the results suggested that MPSE is a potent radiosensitizer against breast cancer cells.

### MPSEs ameliorates radiation-induced EMT process in epithelial breast cancer cells

**Fig. 3:**
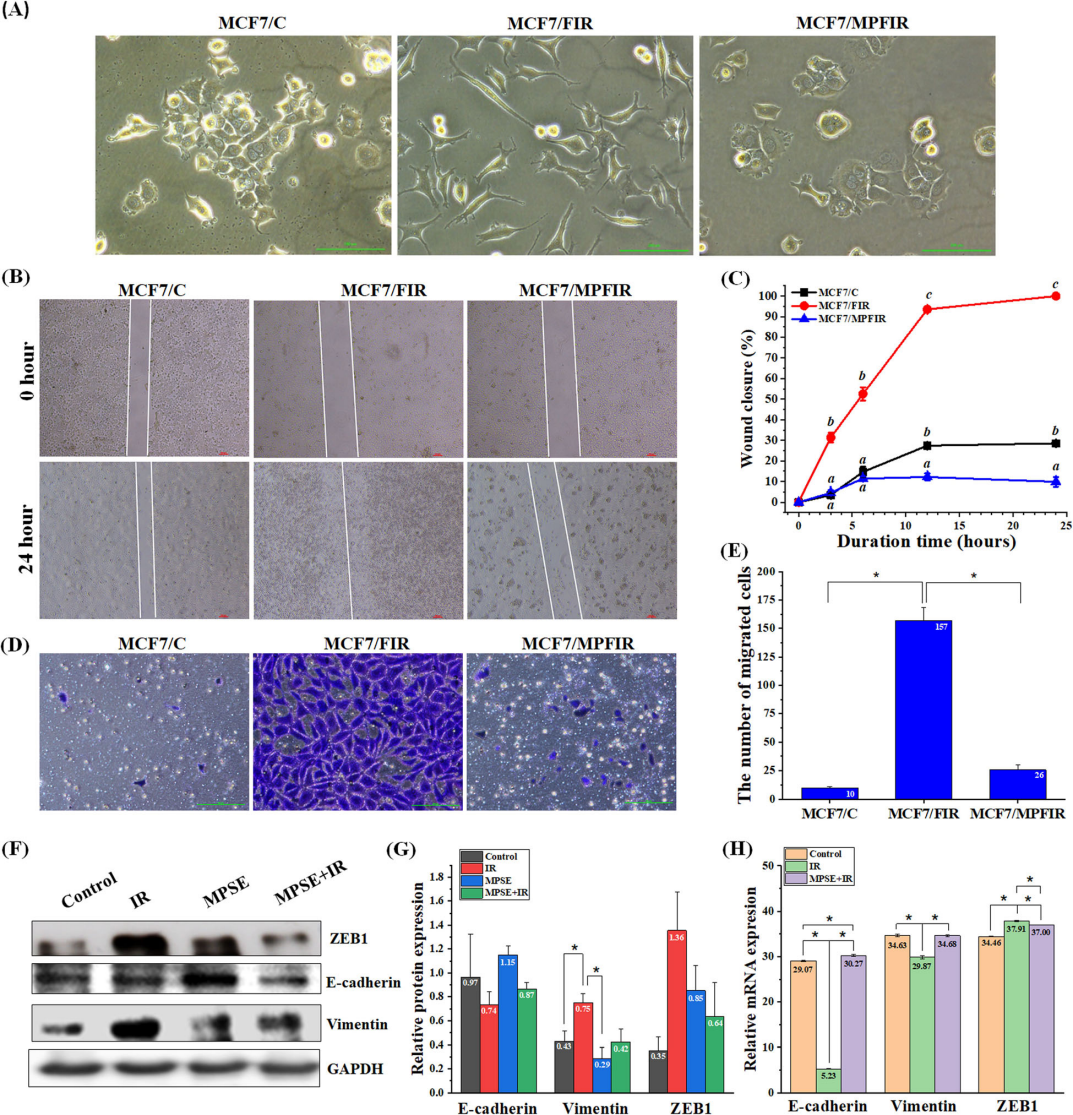
The effect of MPSE on radiation-induced EMT in breast cancer MCF7 cells. **A** Morphological observation showed variance between untreated MCF7 control cells and MCF7 treated with IR alone or in combination with MPSE. **B** A wound healing assay was performed to analyze the migration ability of MCF7 cells; monolayer cells were scratched with a 200 μL micropipette tip. Images were obtained at 0 and 24 h after creating the scratch. **C** Migration potential of breast cancer cell lines was determined by measuring the wound width as a function of time. The statistical significance of each group at various time points was analyzed by a one-way ANOVA and Tukey’s HSD test (different letters represent *p* < 0.05). **D** Representative photographs of transwell migration assay and **E** the number of transmembrane cells. The scale bar represents 100 μm. Data are presented as mean ± SD of three independent experiments (numbers in the bars represent the mean of each group). The statistical significance of each group was analyzed by a one-way ANOVA and Tukey’s HSD test (* *p* < 0.05). **F** Western blot analysis of EMT-related protein and **G** analysis of protein expression. Bar graph shows the relative expressions of proteins normalized to GAPDH. The data are shown as the mean ± SD of three independent experiments (numbers in the bars represent the mean of each group). The statistical significance of each group was analyzed by a one-way ANOVA and Tukey’s HSD test (* *p* < 0.05). **H** EMT-related gene expression was determined by qPCR using HPRT as the loading control. Relative EMT-related gene expression is presented in a bar graph. The data are shown as the mean ± SD of three independent experiments (numbers in the bars represent the mean of each group). The statistical significance of each group was analyzed by a one-way ANOVA and Tukey’s HSD test (* *p* < 0.05)

To explore the possible effect of IR on causing breast cancer to undergo the EMT process. We initially examined the morphological traits and cell migration activity of the survived MCF7 (MCF/FIR) cells following fractionated doses of radiation (3.3 Gy/day × 3 days). Morphological observation showed that the parental MCF7 cells and the MCF/IR cells were differentiated in terms of morphology. The parental MCF7 cells were generally cuboid-shaped with a strong cell-cell adhesion, but most of the MCF7/FIR cells transformed into mesenchymal spindle-shaped cells with increased intercellular distance (Fig. 3A). To investigate whether morphological transformation was attributed to the EMT process, wound healing and transwell migration assays were performed to assess the effect of IR on the migratory and invasive capabilities of treated MCF7 cells. The wound healing assay confirmed the migratory potential of MCF7/FIR and the percentage of wound closed, which was determined as a function of time, showing that MCF7/FIR exhibited the highest migration rate compared to MCF7 (Fig. 3B,C). In addition, the transwell assay showed that MCF7/FIR cells acquired an aggressive malignant phenotype compared with the parental MCF7 cells, as evidenced by the 16-fold higher number of cells migrating through the membrane than that of parental MCF7 cells (Fig. 3D,E), suggesting that radiation is able to induce an aggressive malignant phenotype of MCF7 breast cancer. We next determined the expression of EMT-related protein using qPCR and Western blot analysis. The result demonstrated that the expressions of mesenchymal marker vimentin and ZEB1 were up-regulated, whereas the expression of epithelial marker E-cadherin protein were down-regulated in MCF7 cells exposed to IR in comparison to the parental cells (Fig. 3F-H). These results demonstrated that irradiation induced EMT in MCF7 cells. Surprisingly, pre-treatment of MCF7 cells with MPSE (MCF7/MPFIR) effectively suppressed the IR-induced EMT process. We observed that the breast cancer mesenchymal traits of IR-treated MCF7 cells were suppressed after pre-treatment with MPSE as the cells did not acquire any of the characteristic mesenchymal morphological features (Fig. 3A). The enhanced migratory and invasive properties of MCF7/FIR were also suppressed by MPSE (Fig. 3B-E). Furthermore, MPSE inhibited IR-induced up-regulation of EMT-related protein (Fig. 3F-H). The up-regulation of E-cadherin and down-regulation of vimentin and ZEB1 were observed in MCF7 cells pre-treated with MPSE compared to MCF7 exposed to IR (MCF7/IR). Our findings indicated that MPSE could remarkably suppress irradiation-induced EMT-associated migration and invasion in breast cancer cells.

### MPSE suppressed radiation-induced stemness phenotype of breast cancer cells

**Fig. 4:**
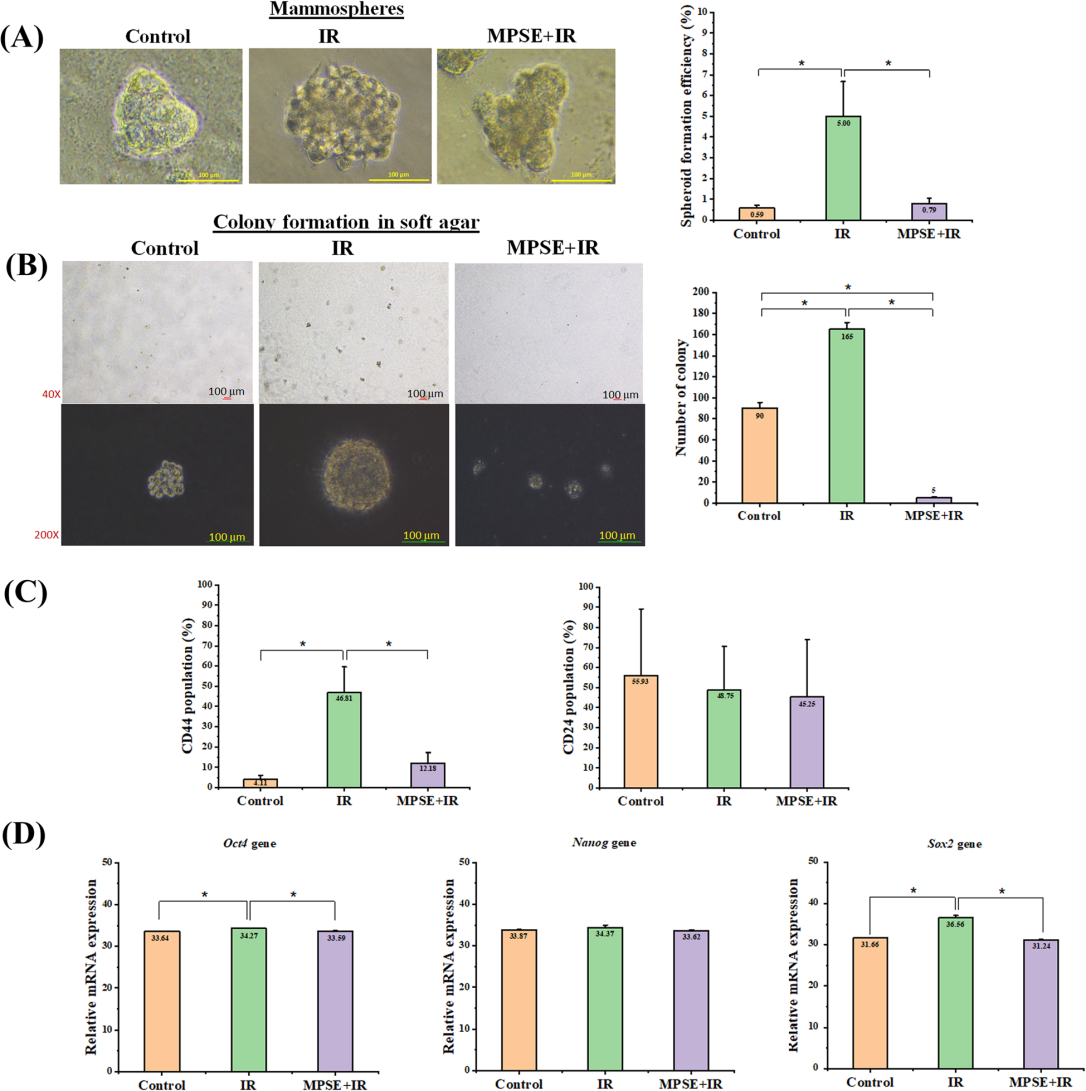
MPSE suppressed radiation-induced CSCs phenotype in a breast cancer cell line. **A** Morphology of mammospheres obtained from fractioned irradiated breast cancer cells and culturing for 10 days. Pictures were taken and mammospheres formation was quantified by counting mammosphere numbers per well on day 10. **B** Representative images of anchorage-independent growth (soft agar) of MCF7 control, MCF7 treated with IR, and MCF7 treated with MPSE+IR cells were grown for 14 days. The upper panel represents the whole number of colonies formed/field, and magnified images are displayed in the lower panel. The scale bar represents 100 μm. The colony number was counted and analyzed. Data are presented as mean ± SD of three independent experiments (numbers in the bars represent the mean of each group). The statistical significance of each group was analyzed by a one-way ANOVA and Tukey’s HSD test (* *p* < 0.05). **C** The percentage of breast cancer cells populations that expressed CSCs markers (CD44, CD24). Data are presented as mean ± SD of three independent experiments (numbers in the bars represent the mean of each group). The statistical significance of each group was analyzed by a one-way ANOVA and Tukey’s HSD test (* *p* < 0.05). **D** CSCs-related gene expression was determined by qPCR using HPRT as a loading control. The relative CSCs-related gene expression is presented in a bar graph. Data are mean ± SD of three independent experiments (numbers in the bars represent the mean of each group). The statistical significance of each group was analyzed by a one-way ANOVA and Tukey’s HSD test (* *p* < 0.05)

To determine the impact of IR on stemness in breast cancer cells, and whether MPSE can inhibit IR-induced stemness, we examined the CSC characteristics of the treated MCF7 breast cancer cells. Breast cancer stem cells (BCSCs) can be identified by their CSC surface markers CD44^+^/CD24^−/low^. Parental MCF7 cells were characterized by CD44^low^/CD24^+^ labeling in flow cytometric analysis; in contrast, after IR treatment, we detected an increasing CD44^+^/CD24^low^ cell population in treated MCF7 cells compared to parental cells (Fig. 4C). The stem cell functional properties of these BCSCs were further confirmed by the in vitro formation of mammospheres and their ability to grow and survive in anchorage-independent growth assay. As expected, a higher number of mammosphere formations and a higher capacity to form colonies in soft agar, which were also more prominent in colony size, were observed in irradiated MCF7 cells compared to untreated MCF7 cells (Fig. 4A, B). In addition, the CSCs-related transcription factors Octamer-binding transcription factor 4 (Oct4), SRY-Box transcription factor 2 (Sox2), and Nanog were expressed in all breast cancer cell lines (Fig. 4D). The expression levels of Sox2, Oct4, and Nanog genes were higher in MCF7 cells treated with IR compared to untreated control MCF7 and MCF7 treatment with combined MPSE/IR (Fig. 4C). The results showed that IR treatment could enhance BCSCs population, as evidenced by CSC surface markers, mammospheres formation and ability to form colonies in soft agar, and the increased mRNA expression levels of CSC markers. In contrast, pre-treatment of MPSE before exposure to IR effectively abolished IR-induced BCSCs (Fig. 4A-D). Together, these data suggested that IR can induce a BSCSs population. However, pre-treatment of MCF7 cells with MPSE before IR can sensitize MCF7 cells to IR through inhibiting IR-induced EMT and CSC formation.

### Proteomic analysis of altered proteins after combined MPSE and IR treatment in breast cancer cells

**Fig. 5:**
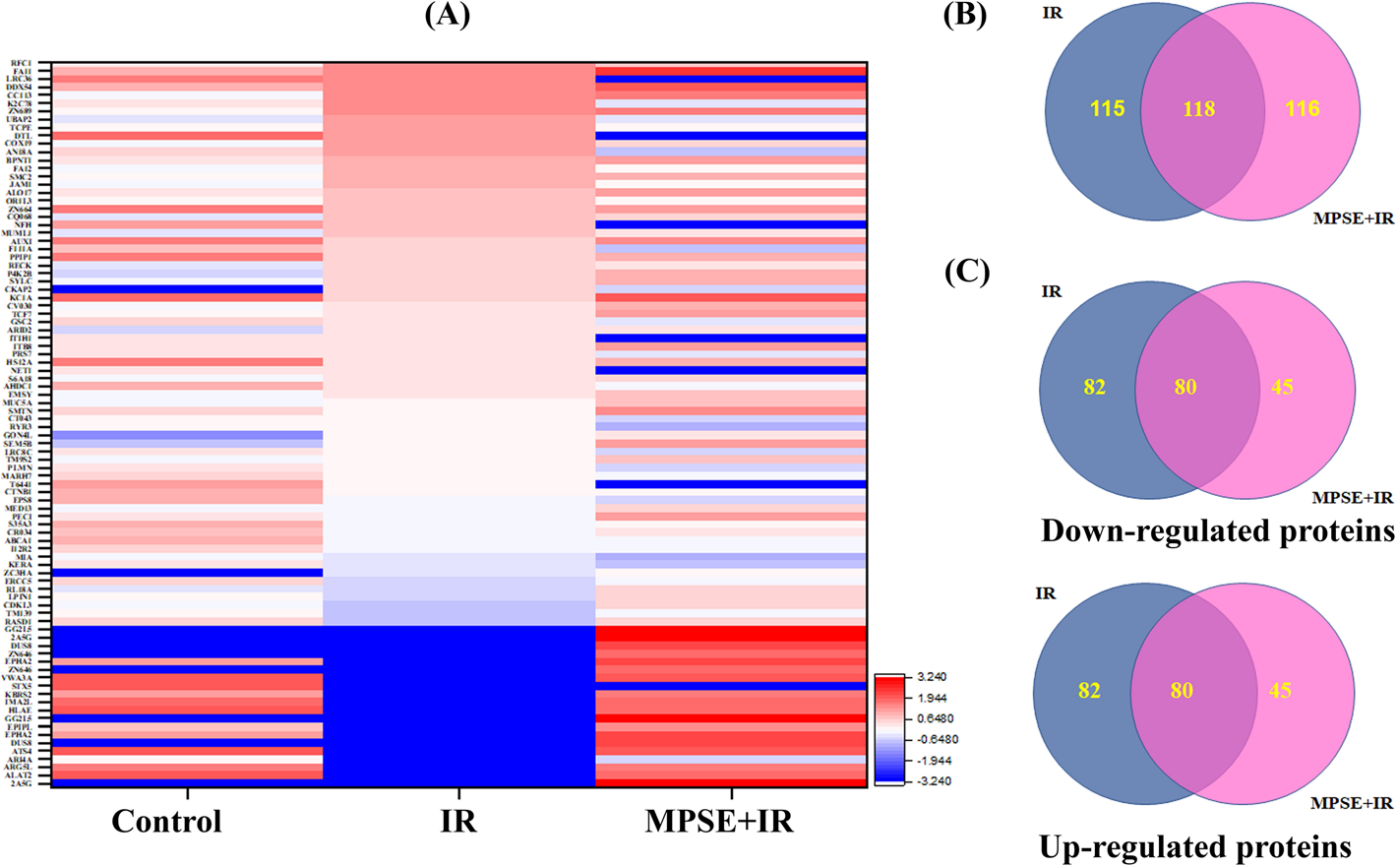
Proteomic analyses of differentially expressed proteins in breast cancer cells after treated with IR alone or combined treatments with IR and MPSE. **A** Heatmap of differentially expressed proteins showing proteins that were differentially expressed in breast cancer cells treated with IR or IR + MPSE compared with control untreated cells. **B** Venn diagram displaying the difference in proteins expressions between IR alone and combined IR with MPSE-treated MCF7 cells. **C** Venn diagram displaying the overlap between the quantified proteins

To investigate the underlying molecular mechanisms of MPSE induced-radio-sensitization, proteomic analysis by LC-MS/MS was performed to identify differentially expressed proteins induced by either IR or the combination of MPSE and IR. A total of 1130 proteins were identified in the data sets. Comparative differential protein-expression analysis using a criterion based on fold change, 1.5-fold or greater, we identified 349 proteins that were differentially expressed, and the distribution of the number of changed proteins is summarized in Fig. 5A,B (The complete lists of identified proteins which were downregulated, upregulated, and not significantly altered by the effects of IR or MPSE+IR in MCF7 cells are in Table S3). A total of 134 proteins (82 up-regulated and 52 down-regulated) were uniquely regulated by IR, and 135 proteins (45 up-regulated, 90 down-regulated) were uniquely regulated by the combination treatment (Fig. 5C).

**Fig. 6:**
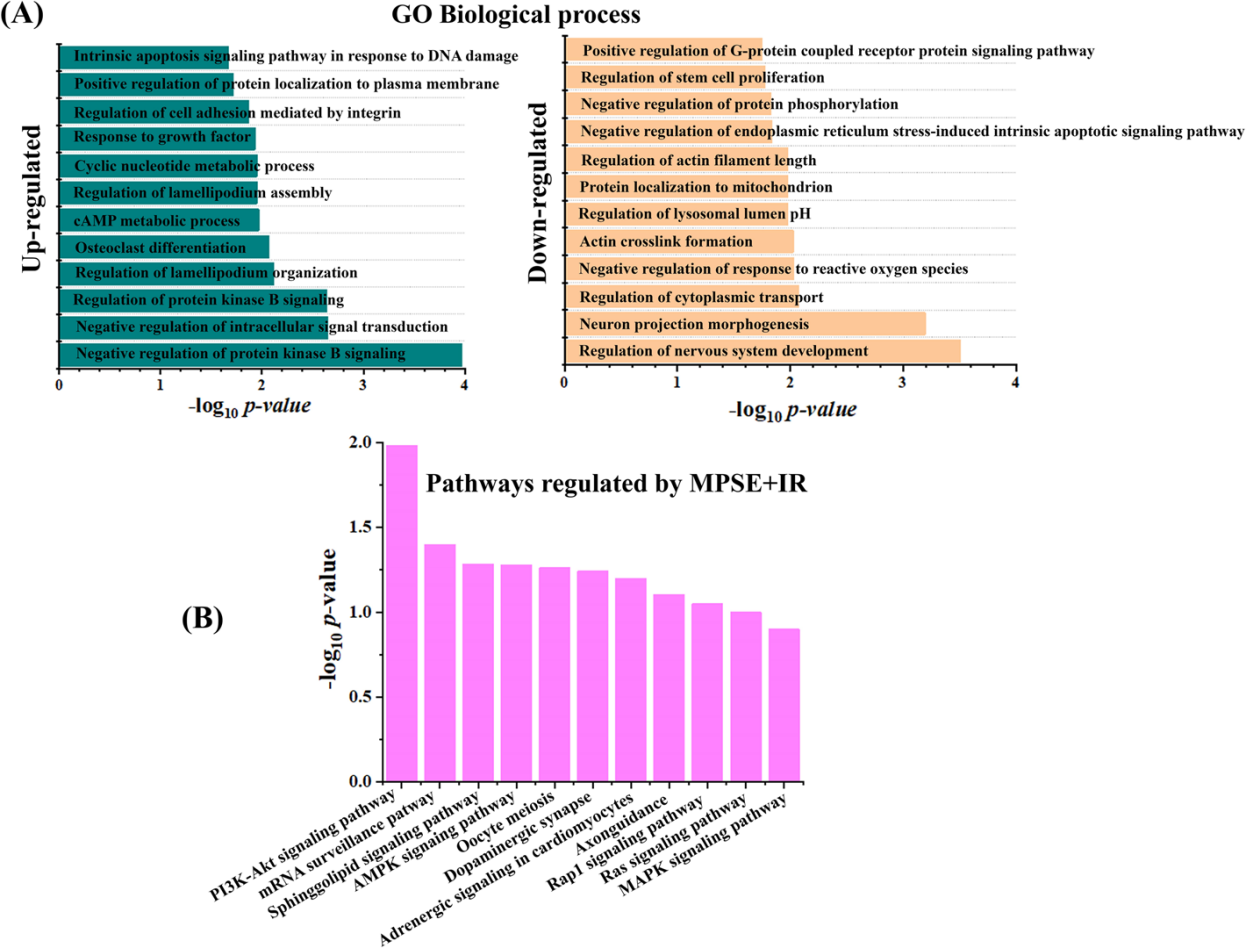
Proteomic analysis of differentially expressed proteins in breast cancer cells after combined treatment with IR and MPSE. **A** Biological Gene Ontology (GO) analyses of the up- and down-regulated protein alteration in MCF7 treated with a combination of MPSE and IR. **B** Ranking of pathways regulated by the combined treated with MPSE and IR based on *p*-values that were identified by KEGG pathway analysis

Next, the up- and down-regulated proteins alteration in MCF7 treated with a combination of MPSE and IR were selected and grouped according to their involvement in specific biological networks using Enrichr software. Gene Ontology (GO) biological process analysis of down-regulated proteins in the combination treatment of MCF7 cells showed multiple cancer-associated pathways and the regulation of stem cell proliferation. Furthermore, the enriched biological processes of up-regulated proteins in combination-treated MCF7 cells are involved in negative regulation of protein kinase B signaling, regulation of cell adhesion, apoptosis induction, cellular stress response, and regulation of phosphatidylinositol 3-kinase signaling (Fig. 6A), suggesting that MPSE treatment in combination with IR is likely to interfere with survival and stemness pathways. To identify the possible signaling pathway with which MPSE may interact, Kyoto Encyclopedia of Genes and Genomes (KEGG) pathway analysis was performed. KEGG pathway analysis revealed that the combination of MPSE and IR modulated key pathways in cancer survival including, the PI3K/AKT signaling pathway, AMP-activated protein kinase (AMPK) signaling pathway, Ras signaling pathway, and MAPK signaling pathway (Fig. 6B). Among these, the PI3K/AKT pathway is the major survival pathway in cancer cells. Active AKT induced by radiation mediates cell survival signals through activation of the DNA damage response (DDR), efficient DNA repair machinery, and suppressing apoptosis.

### Protein–protein interaction networks of selected protein signatures altered by MPSE + IR treatment

**Fig. 7:**
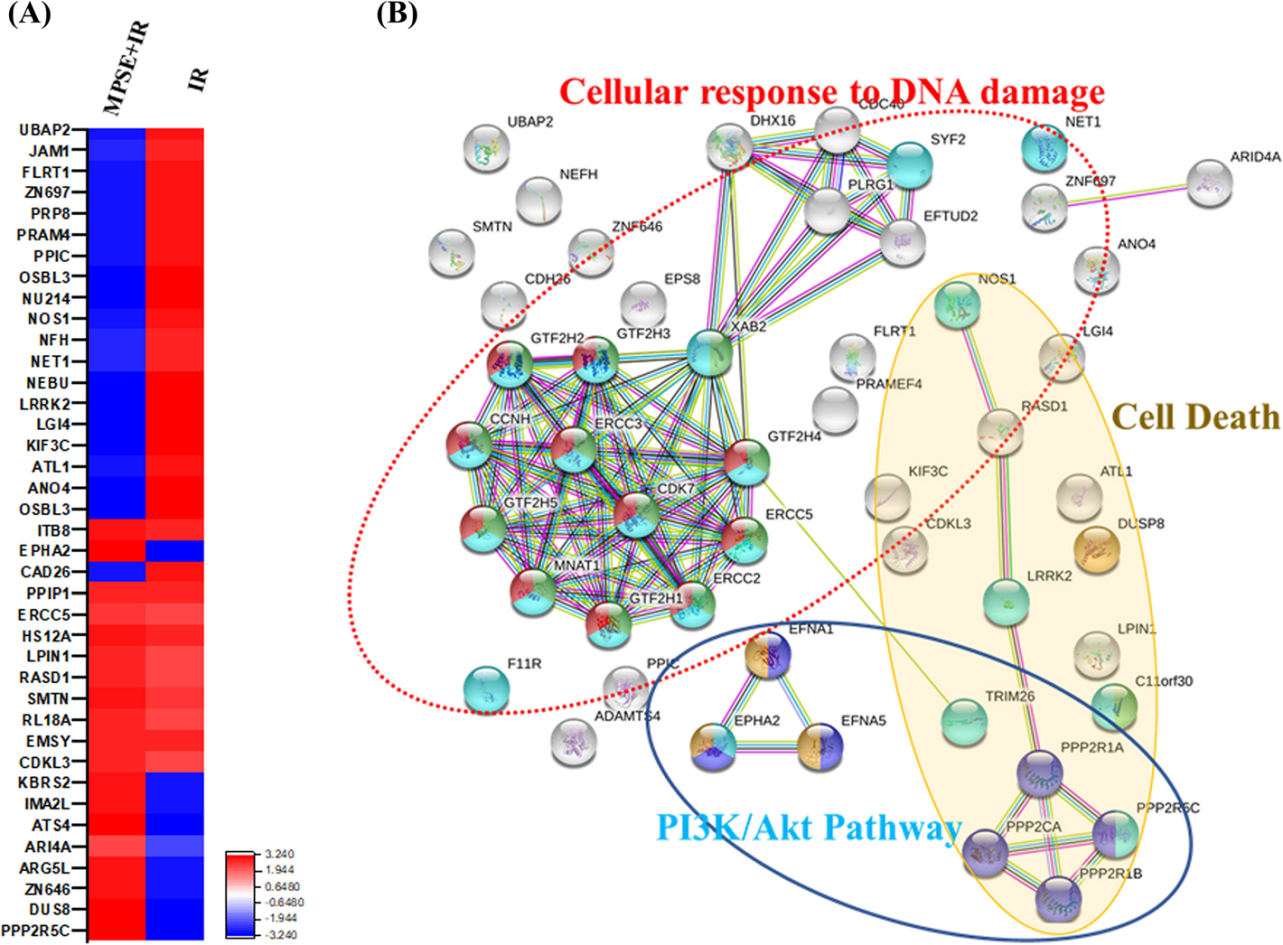
Proteomic analysis of unique differentially expressed proteins by MCF7 breast cancer cell lines after combined treatment with IR and MPSE. **A** Heat map displaying changes in protein levels in MCF7 cells treated with IR, and MCF7 cells combined treated with IR and MPSE; 41 differentially (fold change ≥1.5, *p* < 0.05) expressed proteins in MCF7 cells combined treated with MPSE and IR. **B** Protein network presenting the functional protein–protein interactions of the 41 differentially expressed proteins in MCF7 cells combined treated with MPSE and IR using STRING (v.11.0) software. The three clusters of protein nodes that closely and frequently interacted are indicated by circles. They include proteins involved in the cellular response to DNA damage, apoptosis induction, and PI3K/AKT signaling pathways

To evaluate the unique protein expression and cellular process modulated by these factors after combined MPSE and IR treatment, we compared the list of differentially expressed proteins of MCF7 cells treated with IR and MCF7 cells treated with a combination of MPSE and IR, as shown in Fig. 7A and Table S2. To explain the interaction of 41 significant differentially expressed proteins, the protein-protein interaction (PPI) network based on the STRING database was constructed. The resulting networks are presented in Fig. 7B; a complicated protein–protein interaction network was identified. The main interaction nodes of these proteins were mainly from the three clusters, which separately constituted a related network in response to MPSE and IR treatment-induced damage. One cluster is involved in cellular response to DNA damage including neuroepithelial transforming gene 1 (NET1), excision repair cross-complementing group 5 (ECCR5) and BRCA2-interacting transcriptional repressor EMSY (C11orf30). Another cluster is related to the dephosphorylation of the phosphoinositide 3-kinase (PI3K/AKT) signaling pathway and the induction of apoptotic cell death, including serine/threonine-protein phosphatase 2A (PPP2R5C), leucine-rich repeat kinase 2 (LRRK2), and activator of G-protein signaling 1 (RASD1). Nitric oxide synthase (NOS1) was found to be involved in the response of MCF7 cells to the combination of MPSE and IR treatment. Proteins associated with the EMT process and metastasis were decreased in MCF7 cells treated with the combination of MPSE and IR, including kinesis superfamily protein (KIF3C), leucine-rich glioma inactivated 4 (LGI4), and leucine-rich repeat transmembrane protein (FLRT1).

### MPSE attenuated radiation-induced activation of the PI3K/AKT and MAPK signaling pathways

**Fig. 8:**
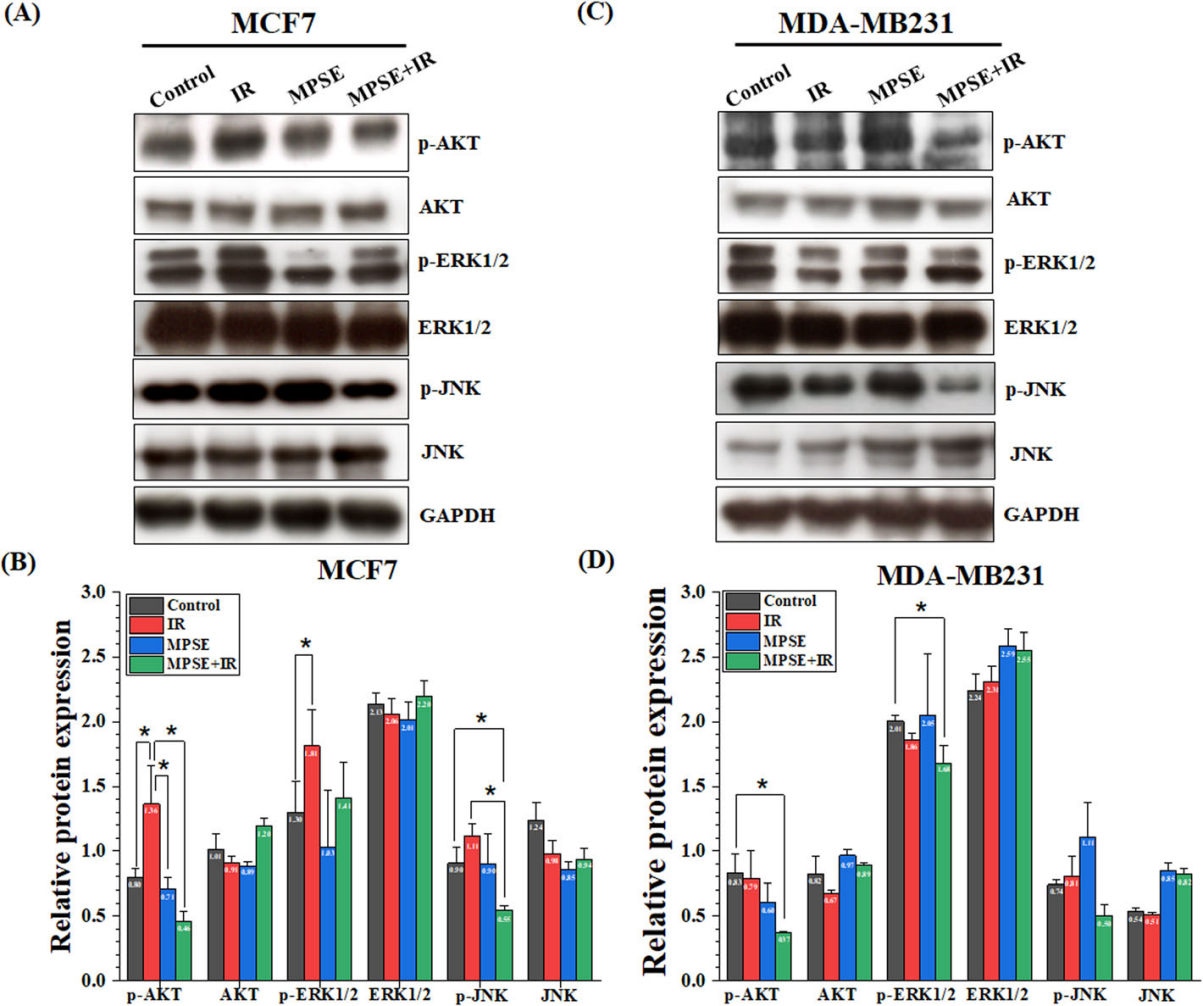
MPSE inhibited radiation-induced activation of the PI3K/AKT and MAPK signaling pathway in breast cancer cells. **A,C** MCF7 and MDA-MB231 cells were pre-treated with MPSE (20 μg/mL) for 24 h and exposed to fractionated irradiation (3.3 Gy/day for 3 days). Total cell lysates were prepared 48 h after treatment and were subjected to Western blotting analysis of PI3K/AKT and MAPK pathways related proteins (total AKT, p-AKT, total ERK1/2, p-ERK1/2, total JNK, and p-JNK) in breast cancer cells. **B,D** Western blot signals were quantified and intensity of phosphorylated and total protein normalized loading control GAPDH are presented in a bar graph. Data are presented as mean ± SD of three independent experiments (numbers in the bars represent the mean of each group). The statistical significance of each group was analyzed by a one-way ANOVA and Tukey’s HSD test (* *p* < 0.05)

To confirm whether pre-treatment with MPSE influences the PI3K/AKT and MAPK pathways in MCF7 cells treated with IR, the key proteins of PI3K/AKT and MAPK pathways, including protein kinase B (AKT), phosphorylated AKT (p-AKT), c-Jun N-terminal kinase (JNK), phosphorylated JNK (p-JNK), extracellular regulated kinases 1/2 (ERK1/2), and phosphorylated ERK1/2 (p-ERK1/2) were determined by Western blot analysis. As shown in Fig. 8A, B, the results revealed that IR alone increases the expression levels of p-AKT, p-ERK1/2, and p-JNK, while there was almost no apparent change in total AKT, total ERK1/2, and total JNK. The combined treatment with MPSE markedly decreased the amount of IR-induced phosphorylation of AKT, ERK1/2, and JNK in MCF7 cells. IR or MPSE alone also increased the expression of p-JNK. In contrast, the expression of p-JNK was strikingly decreased in MCF7 cells after combined treatment with MPSE and IR, while there were no significant expression differences in total JNK. The effect of the combined treatment of MPSE with IR on PI3K/AKT and MAPK pathways was confirmed in other breast cancers, as shown in Fig. 8C,D. In MDA-MB231 triple*-*negative breast cancer, IR did not change the already high basal ERK1/2, JNK, and AKT activation in MDA-MB231 breast cancer cells. In contrast, the combination treatment of MPSE and IR produced the most significant decrease in the phosphorylation of AKT, ERK1/2, and JNK. These results indicated that combined treatment of MPSE with IR regulated the PI3K/AKT and MAPK signaling pathways in breast cancer cells to enhance IR’s efficacy, affecting cell survival, migration, and apoptosis.

## Discussion

Tumor (intrinsic or acquired) radioresistance can be a significant obstacle for the effective treatment of breast cancers. To improve the efficacy of radiotherapy for breast cancer, we evaluated the impact of MPSE, a natural compound (PGG-, EG- and GA-enriched) extracted from *Bouea macrophylla* Griffith seeds known for their anti-cancer activity, on inducing radiosensitization via targeting radiation-induced EMT in breast cancer cells. Our result showed that MPSE might improve the IR effect. The pre-treatment with noncytotoxic dose concentration (IC_20_ = 20 μg/mL) of MPSE before irradiation triggered an increase in cytotoxic effects in breast cancer MCF7 and MDA-MB231 cells in comparison to IR alone. These results indicated a radiosensitization effect of MPSE on breast cancer cells. Next, to explore the possible mechanisms underlying the radiosensitization effect of MPSE. Radiation exposure induces DNA damage through cell cycle arrest, apoptosis, and tumor regression [24]. DNA double-strand breaks (DSBs) induced by radiation are the leading cause of cell death after radiation. Here, we examined the radiation-induced DNA-DSBs by measuring the expression of γH2AX, a DNA double-strand breaks marker [25], by Western blotting and the presence of γH2AX foci using immunocytochemistry. The results showed that IR induced γH2AX foci and γH2AX expression in breast cancer cells. MPSE treatment further increased IR-induced DNA-DSBs, as we observed an increase in γH2AX expression and the presence of γH2AX foci in the combination treatment group. A previous study showed that MPSE promotes intracellular reactive oxygen species (ROS) production and prevents cell cycle progression, leading to inhibition of proliferation and apoptosis induction in breast cancer MCF7 cells [14]. Our results suggested that pre-treatment with MPSE triggers ROS overproduction in IR-treated cells and enhances the effect of radiation on breast cancer cells via increasing IR-induced DNA damage.

Recent studies revealed that radiation could induce increases in cancer stem cell phenotype and aggressive malignant phenotype by undergoing the EMT process related to radioresistance development in radiation-survived cancer cells [26–29]. In recent years, the relationship between the EMT process, cancer stemness, and radiation resistance has gradually attracted scientists’ attention. Herein, our findings confirmed the radiation-induced EMT process; IR-treated MCF7 cells have a mesenchymal phenotype including spindle-liked shape, loss of cell-cell adhesion. And high migration ability accompanied by an expression of ZEB1 and vimentin, and diminished E-cadherin expression. ZEB1 has been reported to play a crucial role in modulating irradiation-induced EMT [30]. A study indicated that B-cell CLL/lymphoma 6 (BCL6) promotes EMT via enhancing the ZEB1-mediated transcriptional repression of E-cadherin in breast cancer cells [31]. Targeted inhibition of ZEB1 increases radiosensitivity in cancer cells through an EMT-dependent or -independent mechanism. It has been reported that non-stem cancer cells that undergo the EMT process are associated with increases in cellular plasticity and stemness [4]. Several studies have reported that the IR-induced EMT program confers CSCs properties [7, 32]. Accordingly, this study reported that IR-treated MCF7 cells exhibited more of a CSC phenotype by displaying several CSC markers such as CD44, activated Sox2, Oct4, and Nanog [33]. IR-treated MCF7 cells significantly expressed a low level of CD24 and high levels of CD44 compared to untreated control MCF7 cells. We also found that pluripotent transcription factors Sox2 and Oct4 were markedly up-regulated in IR-treated MCF7 cells as the ability of the cancer cells to grow and survive in anchorage-independent conditions has been widely accepted as a hallmark of CSCs [34]. Our findings are further supported by the in vitro tumorigenicity assay using the soft agar colony formation assay. We found that IR-treated MCF7 cells had the highest ability to form colonies than control MCF7 cells, indicating a higher tumorigenic potential in vitro. These data suggested that radiation-induced breast CSCs phenotypic characteristics include enhanced the ability of cells to grow and survive in anchorage-independent conditions and mammosphere formation, overexpressed stemness-associated markers, and EMT in IR-treated MCF7 cells. In contrast, MCF7 cells pre-treatment with MPSE before irradiation attenuated the stemness phenotypic characteristics of breast cancer cells, including decreases in the number of mammospheres, down-regulation of stemness- or EMT-associated markers, migratory ability, as well as the reduced ability of cells to form colonies in an anchorage-independent condition.

A previous study showed that MPSE induced apoptosis in breast cancer cells via the mitochondrial pathway [14]. In this study, we found that the combined treatment of MPSE and IR markedly increased the apoptotic effect in breast cancer cells compared with a single treatment of IR or MPSE. IR treatment can damage DNA and promote cell death by apoptosis, senescence, or mitotic catastrophe (MC) [35]. Although a small amount of apoptosis was observed that did not correlate with the survival fraction (Fig. 1A), due to the lost caspase-3 expression, MCF7 cells are resistant to stress-induced apoptosis [36]. Therefore, we further examined cell death pathways in the MCF7 populations, paying attention to cellular senescence. The results revealed an increasing number of senescent cells in the combination-treated cells compared to cells treated with IR alone (Fig. 2C,D). Cellular senescence was suggested to play a crucial role in the regression of tumors exposed to IR [37–39]. Several studies showed that cancer cells might become prematurely senescent rather than apoptotic in response to DNA damage depending on the cellular context [39, 40]. This result suggests that pre-treatment with MPSE enhanced the effect of IR-induced apoptosis, and senescence cell death is one of the mechanisms of radio-sensitization in breast cancer cells. Taken together, the results suggest that MPSE pre-treatment attenuates the IR-induced stemness phenotype and EMT of breast cancer cells, increased the effect of IR-induced DNA damage, and promotes cell death, leading to decreased cell survival in irradiated breast cancer cells, therefore supporting the use of MPSE as a radiosensitizer in the multimodal treatment of breast cancer.

To further explore the action mechanism of this new formulation of mixed polyphenol (MPSE) in producing a radiosensitivity effect on breast cancer cells and as studies of MPSE are scarce, a proteomic analysis to identify the most relevant protein networks involved in the MPSE effect was investigated. Using this procedure, we were able to retrieve the most affected cellular pathways in response to the combination of MPSE and IR treatment. The essential proteins involved in the negative regulation of the PI3K/AKT and MAPK pathways, including protein phosphatase 2A (PP2A), dual-specificity phosphatase-8 (DUSP-8), AT-rich interactive domain-containing protein 1A (ARID1A), were significantly increased in the combined MPSE + IR-treated cells (Fig. 3A,B). A study revealed that downregulation of phosphatase 2A (PP2A) activity is associated with AKT and ERK activation and promotes LNCaP cells’ growth under androgen-deprived conditions [41]. More importantly, the loss of PPP2CA promotes prostate cancer’s invasiveness due to the activation of AKT/β-catenin signaling pathways and induced EMT [42]. Furthermore, the tumor suppressor gene AT-rich interactive domain-containing protein 1A (ARID1A) plays a vital role in regulating the PI3K/AKT pathway. ARID1A mutation in cancer was found to occur synergistically with PIK3CA [43]. The up-regulation of phosphorylated AKT was observed in cancer cell silencing of ARID1A activation, and restoration of ARID1A inhibited cell proliferation and induced apoptosis [44, 45]. The silencing of ARID1A increased the migration and invasion abilities of the liver cancer cells [46]. Consistently, our proteomic results showed the suppression of critical proteins in the PI3K-mediated AKT-pathway, such as PP2A, DUSP8, and ARID1A, which were simultaneously overexpressed in breast cancer cells treated with combined MPSE and IR. Moreover, it is well known that cancer stem cells gain the enhanced DNA repair capacity, rendering them resistant to radiation [47]. Notably, our findings showed that pre-treatment with MPSE mediated down-regulation of DNA repair proteins such as DNA excision repair protein (ERCC5) [48], neuroepithelial cell-transforming gene 1 protein (NET1) [49], BRCA2-interacting transcriptional repressor (EMSY) [50], and junctional adhesion molecule A (F11R), which enhanced the radiosensitivity of breast cancer cells by reducing the formation of cancer stem cells. These findings are consistent with our earlier results (γH2AX), which revealed that the combination treatment of MPSE with IR enhances the efficacy of IR by increasing IR-induced DNA damage and IR-induced cell death. The treatment of MCF7 with IR + MPSE suppressed the IR-induced EMT process in breast cancer cells. Additionally, most of the phosphatase proteins modulating the PI3K/AKT and MAPK pathways showed increased expressions in the combination treatment group compared to the IR alone group. Accordingly, the PI3K/AKT pathway was shown to be involved in radioresistance and enhancing CSC phenotypes in irradiated cancer cells [51]. MAPK pathways play an importance regulatory role in cell proliferation, migration, metastasis, and apoptosis [52]. This led us to the next confirmation analysis to determine the effect of the combination of MPSE and IR on regulating the activation of the PI3K/AKT and MAPK pathways, which might result in the improvement of radiotherapy and in overcoming radioresistance.

Pre-treatment with MPSE before IR enhancing the radio-sensitization was also associated with a marked decrease in the IR-induced phosphorylation of AKT, ERK1/2, and JNK in MCF7 and MDA-MB231 cells compared to cells treated with IR alone (Fig. 8A, B). The MAPK and PI3K/AKT pathways are activated in irradiated cancer cells and regulate fundamental cellular processes associated with radioresistance, including proliferation, apoptosis, and metastasis [51]. A literature review suggested that these survival pathways activated in response to irradiation could be an appropriate target to overcome radioresistance in breast cancer radiotherapy [53, 54]. Evidence supports that radiation increases phosphorylated AKT1 and AKT2 in the breast cancer MCF7 mammospheres CD44+/CD24−/low-expressing cells, whereas the inhibition of the AKT signaling pathway sensitizes MCF7 mammosphere cells to ionizing radiation [55]. Yuan et al. reported that ionizing radiation (IR) could promote tumor metastasis by activation of B-lymphoma Moloney murine leukemia virus insertion region-1 (Bmi1)-regulated radiation-induced EMT via activation of PI3K/AKT signaling in breast cancer cells [56]. Radiation also induces the activation of the PI3K/AKT signaling pathway in breast cancers and targeting this pathway by the dual PI3K/mTOR inhibitor NVP-BEZ235 sensitizes the MDA-MB231 and MCF7 breast cancer cells to IR [57]. Our results suggested that MPSE targets survival pathways in irradiated cancer cells possibly by suppressing phosphorylated ERK1/2, AKT, and JNK, which might be associated with the enhanced radiosensitivity of breast cancer cells by MPSE.

## Conclusion

The present findings suggest that pre-treatment with MPSE produces dramatic radiosensitization of breast cancer cells in vitro and attenuates the IR-induced EMT process and the cancer stemness phenotype. The possible molecular mechanisms might be involved with the suppression of phosphorylated ERK1/2, AKT, and JNK. In summary, our study provides a novel strategy for increasing the efficacy of radiotherapy in breast cancer patients using a safer and low-cost natural product, MPSE.

## Supplementary Information

**Additional file 1: Table S1.** Differential expressed proteins among MCF7 treated combination of MPSE and IR compared to MCF7 treated IR alone. Proteins specific to one of the two groups compared were assigned a fold change of infinity. **Table S2.** List of primer sequences used in quantitative PCR assays. **Figure S1.** The uncropped Western blot images corresponding to Fig. 1D showing all the bands. Red boxes indicate the samples of interest. **Figure S2.** The uncropped Western blot images corresponding to Fig. 3F showing all the bands. **Figure S3.** The uncropped Western blot images of (A) MCF7 cells (left panel) and (B) MDA-MB231 cells (right panel) showing all the bands corresponding to Fig. 8A and C, respectively. Red boxes indicate the samples of interest. Black box indicates the Western blot images of interested t-AKT, t-ERK1/2 and t-JNK in (A) MCF7 cells and (B) MDA-MB231 cells are from the same membrane.

**Additional file 2: Table S3.** The overview of protein identifications and quantifications for MCF7 cells treated with IR or IR + MPSE. **Table S4.** qRT-PCR raw-data for the EMT-related gene (Article Fig. 3) and CSCs-related gene (Article Fig. 4).

## Data Availability

The datasets used and/or analyzed during the current study is available from the corresponding author on request.

## Abbreviations

BCSCs: Breast cancer stem cells
bFGF: Basic fibroblast growth factor
BSA: Bovine serum albumin
DTT: Dithiothreitol
EG: Ethyl gallate
EGF: Epidermal growth factor
EMT: Epithelial–mesenchymal transition
ERK1/2: Extracellular signal-regulated kinase 1/2
FBS: Fetal bovine serum
FITC: Fluorescein isothiocyanate
GA: Gallic acid
GADPH: Glyceraldehyde 3-phosphate dehydrogenase
Gy: Gray
IR: Irradiation
CSC: Cancer stem cell
JNK: C-Jun N-terminal kinase
KEGG: Kyoto encyclopedia of genes and genomes
LC-MS/MS: Liquid chromatograph-mass spectrometer
mRNA: Messenger ribonucleic acid
MAPK: Mitogen-activated protein kinase
MCF7/C: MCF7 cells untreated control group
MCF7/FIR: MCF7 cells survive treated fractionated irradiation
MCF7/MPFIR: MCF7 cells survive a treated with MPSE and fractionated irradiation
MPSE: Maprang seed extract
Oct4: Octamer-binding transcription factor 4
PGG: 1,2,3,4–6-pentyl-O-galloyl-β-d-glucose
PI3K/AKT: Phosphatidylinositol-3-kinase/protein kinase B
PMSF: Phenylmethylsulfonyl
SA*-*β-*G*al: Senescence-associated-β-galactosidase
SF: Surviving fraction
SERs: Sensitization enhancement ratios
SOX2: SRY-Box transcription factor 2
