# Supplementary material for: A radiosensitizer, gallotannin-rich extract from Bouea macrophylla seeds, inhibits radiation-induced epithelial-mesenchymal transition in breast cancer cells
Source: BMC Complement Med Ther. 2021 Jul 3;21:189. doi: 10.1186/s12906-021-03363-6 (PMC8254241; doi:10.1186/s12906-021-03363-6)
Supplement: Supplementary file 1 — Additional file 1: Table S1. Differential expressed proteins among MCF7 treated combination of MPSE and IR compared to MCF7 treated IR alone. Proteins specific to one of the two groups compared were assigned a fold change of infinity. Table S2. List of primer sequences used in quantitative PCR assays. Figure S1. The uncropped Western blot images corresponding to Fig. 1D showing all the bands. Red boxes indicate the samples of interest. Figure S2. The uncropped Western blot images corresponding to Fig. 3F showing all the bands. Figure S3. The uncropped Western blot images of (A) MCF7 cells (left panel) and (B) MDA-MB231 cells (right panel) showing all the bands corresponding to Fig. 8A and C, respectively. Red boxes indicate the samples of interest. Black box indicates the Western blot images of interested t-AKT, t-ERK1/2 and t-JNK in (A) MCF7 cells and (B) MDA-MB231 cells are from the same membrane. [file 12906_2021_3363_MOESM1_ESM.docx]

*Supplementary Information*

A radiosensitizer, gallotannin-rich extract from *Bouea macrophylla* seeds, inhibits radiation-induced epithelial-mesenchymal transition in breast cancer cells.

Jiraporn Kantapan ^1^*, Siwaphon Paksee ^1^, Aphidet Duangya ^2^, Padchanee Sangthong ^3,4^, Sittiruk Roytrakul ^5^, Sucheewin Krobthong ^6^, Wipob Suttana^7^, and Nathupakorn Dechsupa ^1^

^1^ Molecular Imaging and Therapy Research Unit, Department of Radiologic Technology, Faculty of Associated Medical Sciences, Chiang Mai University, Chiang Mai 50200, Thailand.

^2^ Interdisciplinary Program of Biotechnology, Graduate School, Chiang Mai University, Chiang Mai 50200, Thailand.

^3^ Department of Chemistry, Faculty of Science, Chiang Mai University, Chiang Mai 50200, Thailand.

^4^ Research Center on Chemistry for Development of Health Promoting Products from Northern

Resources, Chiang Mai University, Chiang Mai 50200, Thailand.

^5^ Functional Ingredients and Food Innovation Research Group, National Center for Genetic Engineering and Biotechnology (BIOTEC), National Science and Technology Development Agency (NSTDA), Pathum Thani 12120, Thailand.

^6^ National Omics Center (NOC), National Science and Technology Development Agency (NSTDA), Pathum Thani 12120, Thailand.

^7^ Department of Biomedical Science, School of Health Science, Mae Fah Luang University, Chiang Rai 57100, Thailand.

***** Corresponding author:

Jiraporn Kantapan

Department of Radiologic Technology,

Faculty of Associated Medical Sciences,

Chiang Mai University, Chiang Mai 50200, Thailand

e-mail: [jiraporn.kan@cmu.ac.th](mailto:jiraporn.kan@cmu.ac.th); Tel.: +665-393-6022

**Supplementary Table S1.** Differential expressed proteins among MCF7 treated combination of MPSE and IR compared to MCF7 treated IR alone. Proteins specific to one of the two groups compared were assigned a fold change of infinity.

| **Gene** | **Full Name** | **Log_2_ FC (MPSE+IR *vs* IR)** | **Function** |
| --- | --- | --- | --- |
|  |  |  |  |
| PPP2R5C | Serine/threonine-protein phosphatase 2A 56 kDa regulatory subunit gamma isoform | ∞ | ERK dephosphorylation, activate TP53 and play a role in DNA damage-induced inhibition of cell proliferation |
| DUSP8 | Dual Specificity Phosphatase 8 | ∞ | Dephosphorylation activity against JNK |
| ZN646 | Zinc Finger Protein 646 | ∞ | May be involved in transcriptional regulation. |
| ARG5L | Rho guanine nucleotide exchange factor 5-like protein | ∞ | Regulation of cell shape and actin cytoskeletal organization |
| ARID4A | AT-rich interactive domain-containing protein 4A | ∞ | Metastasis suppressor |
| ADAMTS4 | ADAM Metallopeptidase with Thrombospondin Type 1 Motif 4 | ∞ | Antiangiogenic protein |
| IMA2L | Importin subunit alpha-2-like protein | ∞ | Nuclear export protein that are involved in cell proliferation, differentiation, DNA repair, and tumorigenesis |
| KBRS2 | NF-kappa-B inhibitor-interacting Ras-like protein 2 | ∞ | Negatively regulating both the NF-kB pathways |
| CDKL3 | Cyclin-dependent kinase-like 3 | 1.63 | Involved in cell cycle control |
| EMSY | BRCA2-interacting transcriptional repressor EMSY | 0.70 | DNA repair |
| RL18A | 60S ribosomal protein L18a | 1.51 | RNA binding |
| SMTN | Smoothelin | 1.60 | Actin cytoskeletal organization |
| RASD1 | Dexamethasone-induced Ras-related protein 1 | 1.73 | Nitric oxide mediated signal transduction, suppress cell proliferation |
| LPIN1 | Phosphatidate phosphatase LPIN1 | 1.55 | Lipid metabolism |
| HSPA12A | Heat shock 70 kDa protein 12A | 1.08 | Cellular response to stress |
| ERCC5 | DNA repair protein complementing | 0.46 | Nucleotide-excision repair |
| PPIP1 | Proline-serine-threonine phosphatase-interacting protein 1 | 0.45 | Regulation of the actin cytoskeleton |
| EPHA2 | Ephrin type-A receptor 2 | 3.08 | Cell adhesion, apoptotic signaling pathway in response to DNA damage |
| ITB8 | Integrin beta-8 | 1.23 | Cell adhesion |
| CDH26 | Cadherin-like protein 26 | -∞ | Cell polarization and migration |
| OSBL3 | Oxysterol-binding protein-related protein 3 | -∞ | Regulation of the actin cytoskeleton, cell polarity and cell adhesion |
| ANO4 | Anoctamin-4 | -∞ | Ca^2+^-dependent ion channel activity |
| ATL1 | ADAMTS-like protein 1 | -∞ | Migration and invasion |
| KIF3C | Kinesin-like protein KIF3C | -∞ | Metastasis |
| LGI4 | Leucine-rich repeat LGI family member 4 | -∞ | Metastasis |
| LRRK2 | Leucine-rich repeat serine/threonine-protein kinase 2 | -∞ | Migration and proliferation |
| NEBU | Nebulin | -∞ | Actin binding protein |
| NET1 | Netrin-1 | -∞ | Anti-apoptosis |
| NFH | Neurofilament heavy polypeptide | -∞ | Cell projection assembly |
| NOS1 | Nitric oxide synthase | -∞ | Modulate DNA repair mechanisms by upregulating p53 and the DNA-dependent protein kinase (DNA-PK). |
| NU214 | Nuclear pore complex protein Nup214 | -∞ | Protein transport |
| OSBL3 | Oxysterol-binding protein-related protein 3 | -∞ | Regulation of the actin cytoskeleton, cell polarity and cell adhesion |
| PPIC | Peptidyl-prolyl cis-trans isomerase C | -∞ | Protein folding |
| PRAMEF4 | PRAME family member 4 | -∞ | Anti-apoptosis |
| PRP8 | Pre-mRNA-processing-splicing factor 8 | -∞ | Pre-mRNA splicing |
| ZNF697 | Zinc finger protein 697 | -∞ | Transcription regulation |
| FLRT1 | Leucine-rich repeat transmembrane protein FLRT1 | -∞ | FGFR1-mediated activation of MAP kinases |
| JAM1 | Junctional adhesion molecule A | -∞ | Metastasis |
| UBAP2 | Ubiquitin-associated protein 2 | -∞ | Cancer cell proliferation |
| TRIM26 | Midline-2 | -∞ | Cancer cell proliferation |
| EPS8 | Epidermal growth factor receptor kinase substrate 8 | -∞ | Migration and invasion |

**Supplementary Table S****2.** List of primer sequences used in quantitative PCR assays.

| Primer name | Forward sequence (5’ – 3’) | Reverse sequence (5’ – 3’) |
| --- | --- | --- |
| *CDH1* | CCTTCCTCCCAATACATCTCC | GATTTTGTAGTCACCCACCTC |
| *VIM* | CTCTTCCAAACTTTTCCTCCC | GTTTCGTTGATAACCTGTCCATC |
| *ZEB1* | TTCACAGTGGAGAGAAGCCA | GCCTGGTGATGCTGAAAGAG |
| *Nanog* | GGCAAACAACCCACTTCTGC | GCATCTGCTGGAGGCTGAG |
| *Sox2* | GAGGGGTGCAAAAGAGGAGAG | GCTGTCATTTGCTGTGGGTG |
| *Oct4* | CGCAAGCCCTCATTTCACCAG | CCCATCACCTCCACCACCTG |
| *HPRT* | CCTGGCGTCGTGATTAGTGA | AGGGCTACAATGTGATGGCC |


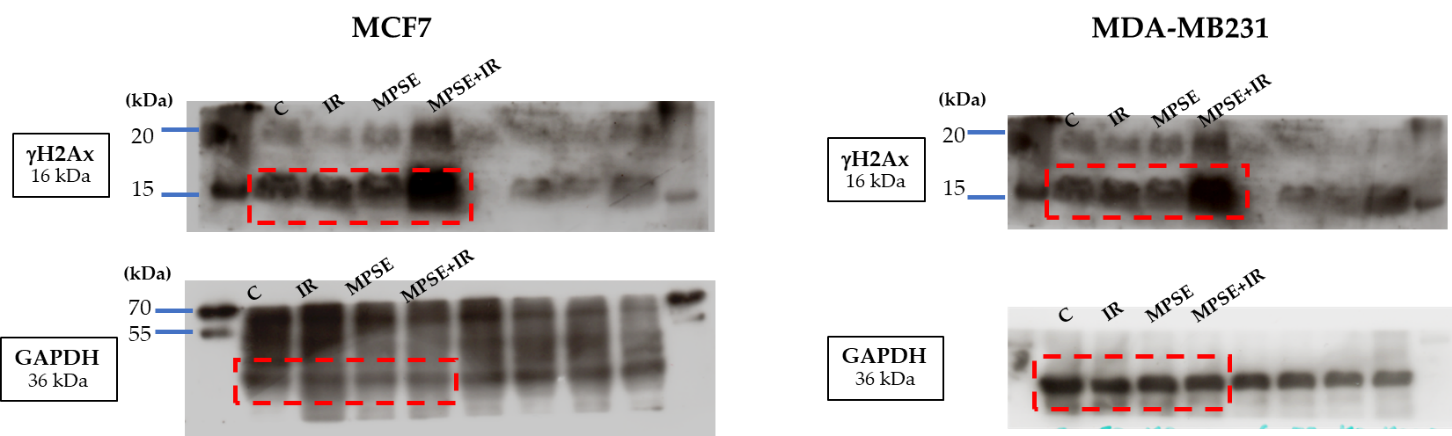


**Supplementary** **Figure S1.** The uncropped Western blot images corresponding to Fig. 1D showing all the bands. Red boxes indicate the samples of interest.


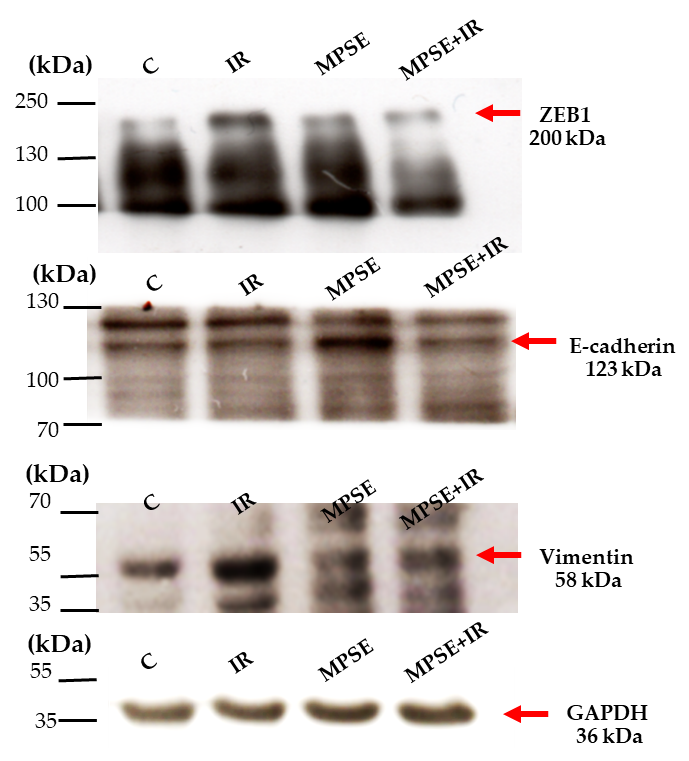


**Supplementary** **Figure S2.** The uncropped Western blot images corresponding to Fig. 3F showing all the bands.

| 1. MCF7 Cells | 1. MDA-MB231 Cells |
| --- | --- |
| 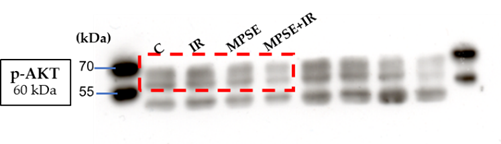 | 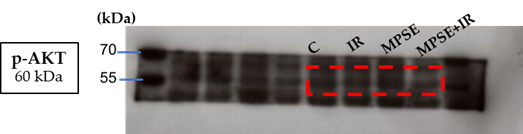 |
| 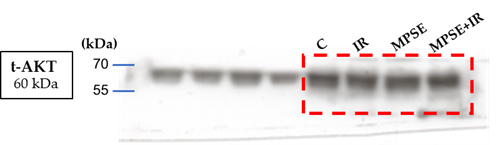 | 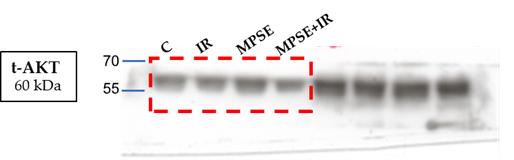 |
| 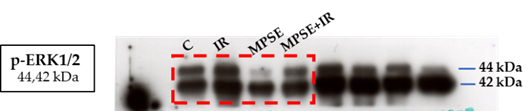 | 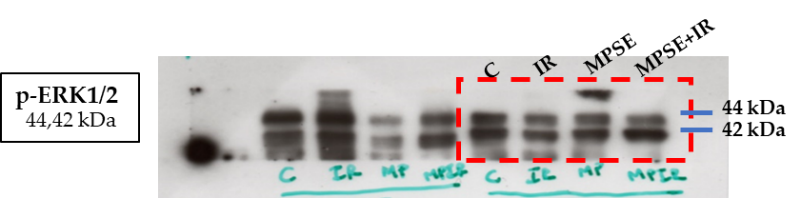 |
| 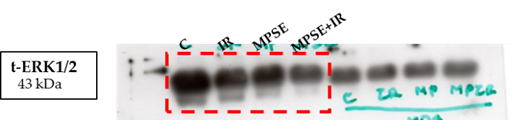 | 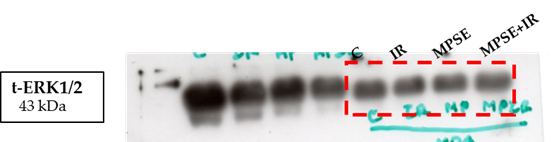 |
| 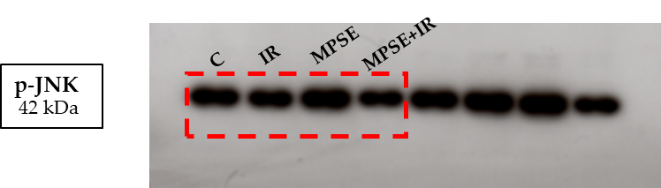 | 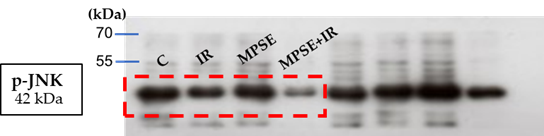 |
| 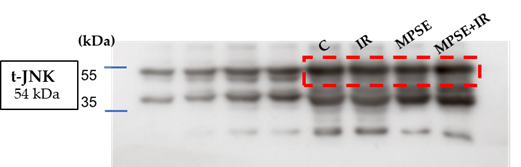 | 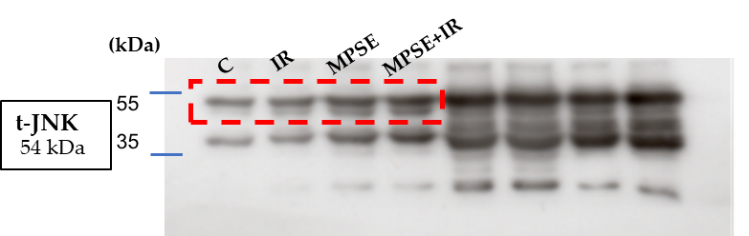 |
| 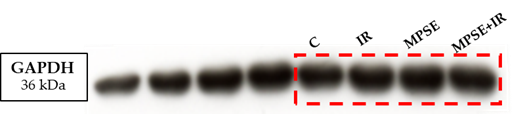 | 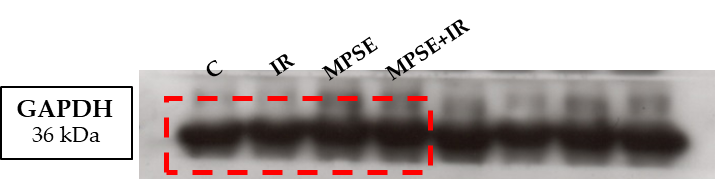 |

**Supplementary** **Figure S3.** The uncropped Western blot images of (A) MCF7 cells (left panel) and (B) MDA-MB231 cells (right panel) showing all the bands corresponding to Fig. 8A and Fig. 8C, respectively. Red boxes indicate the samples of interest. Black box indicates the Western blot images of interested t-AKT, t-ERK1/2 and t-JNK in (A) MCF7 cells and (B) MDA-MB231 cells are from the same membrane.
